# The directionality of collective cell delamination is governed by tissue architecture and cell adhesion in a *Drosophila* carcinoma model

**DOI:** 10.1016/j.isci.2025.113663

**Published:** 2025-09-30

**Authors:** Marta Mira-Osuna, Steffen Plunder, Eric Theveneau, Roland Le Borgne

**Affiliations:** 1 Institut de Génétique et Développement de Rennes (IGDR), Université de Rennes, CNRS UMR 6290, 35000 Rennes, France; 2 Molecular, Cellular and Developmental Biology Department (MCD), Centre de Biologie Integrative (CBI), University of Toulouse, CNRS, UPS, 31062 Toulouse, France; 3 Institute for the Advanced Study of Human Biology (ASHBi), Kyoto University Institute for Advanced Study, Kyoto University, Yoshida-Konoe-cho, Sakyo-ku, Kyoto 606-8501, Japan

**Keywords:** Cancer, Cell biology

## Abstract

Epithelial cells contact each other and the extracellular matrix via cell junctions, establishing mechanochemical barriers. During collective delamination, epithelia-derived tumors detach from the tissue with a directionality that dictates their malignancy. How cell junctions contribute to this process and how the directionality of delamination is regulated remains unknown. We used the *Drosophila Ras*^*V12*^ carcinoma model and found that the loss of septate junctions in epithelial cells triggers apoptosis, whereas in *Ras*^*V12*^-cells promotes collective delamination. We found that apical and basal delamination differ in cell identity, polarity, and junctional remodeling, occurring exclusively in squamous and pseudostratified epithelia, respectively. We performed mathematical simulations using a 2D agent-based model and found that tissue architecture and preferential adhesion between mutant cells and with wild-type neighbors regulate the directionality of delamination. Apical delamination initiates with cells constricting apically, emitting apical protrusions, and forming an apical neck via preferential adhesion to collectively detach from the tissue.

## Highlights

• Loss of septate junction integrity promotes apoptosis
• Loss of septate junction integrity in *Ras*^*V12*^-cells promotes delamination
• Junctional remodeling and cell identity differ between apical and basal delamination
• Preferential adhesion and tissue architecture regulate the directionality of delamination

## Graphical abstract

**Figure:**
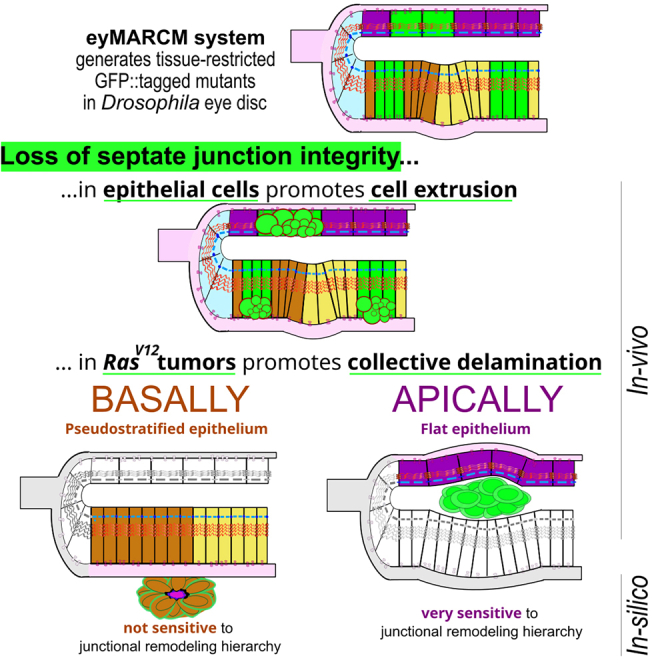

## Introduction

Epithelia are organ-forming tissues with one or multiple layers of polarized cells, connected to each other and the matrix whenever present via cell junctions, that establish mechanochemical barriers and compartmentalize the organism.^1^^,^^2^^,^^3^^,^^4^^,^^5^^,^^6^ Genetic insults in epithelial cells can give rise to carcinomas, which comprise >80% of human cancers. During tumor progression, carcinomas delaminate and detach from the epithelium with a directionality that impacts their fate and malignancy. Tissue surveillance mechanisms remove potentially hazardous cells from the epithelium via apical extrusion in vertebrates and basal extrusion in invertebrates.^7^^,^^8^^,^^9^^,^^10^^,^^11^ However, mutations blocking cell death, such as those in the oncogene Ras (*Ras*^*V12*^), can synergize with genetic insults and promote tumor progression,^12^^,^^13^ by reverting the directionality of delamination and heightening malignancy.^14^^,^^15^ The molecular regulators controlling delamination start to be elucidated^16^^,^^17^^,^^18^ but how the directionality of delamination is controlled and how cell junctions are remodeled during delamination remains poorly characterized. Two types of cell junctions ensure a mechanical connection with the actin cytoskeleton. Adherens junctions (AJ) are formed by complexes comprised of the homophilic adhesion protein E-cadherin (E-cad) connected to the contractile actomyosin network via Catenins.^19^^,^^20^ AJ provides intercellular adhesion and mechanically couples neighboring cells to establish the mechanical barrier of the epithelium. Focal adhesions (FA) are located basally and mediate mechano-chemical signaling with the extracellular matrix via heterodimeric integrin receptors coupled to the basal actin cytoskeleton.^21^ A third type of junctions, septate junctions in invertebrates and tight junctions in vertebrates, ensure the para-cellular diffusion barrier. Septate junctions (SJ) are located basal to AJ, and core-complex components include Coracle (Cora) and Nervana 2 (Nrv2), at bicellular SJ, and Anakonda (Aka), Gliotactin (Gli), and M6 at tricellular SJ. SJ mature during late embryogenesis and establish a functional permeability barrier.^22^^,^^23^^,^^24^^,^^25^ Aka, Gli and M6 assemble at tricellular SJ and anchor bicellular SJ, ensuring overall SJ homeostasis.^26^^,^^27^ Cora and Nrv2 are required for the localization of SJ-core component Sinuous,^28^ which, together with other Claudin-like proteins, also SJ-core components, Kune-Kune and Megatrachea, exert non-occluding functions during trachea size control.^29^ Tight junctions (TJ) locate apical to AJ and are composed of core-components that include Claudins, which share sequence homology with aforementioned SJ core-components.^30^ Claudin 2 prevents tumor progression in human osteosarcoma cells^31^ and Claudins appear mutated in numerous cancers,^32^ promoting malignant transformation and metastasis.^33^^,^^34^ Tricellular TJ has also been observed to be involved in human cancers,^35^^,^^36^^,^^37^ arguing in favor of a role for TJ during tumorigenesis. However, the exact contribution of TJ or SJ during tumorigenesis remains poorly characterized. In *Drosophila*, the loss of M6 in conjunction with *Ras*^*V12*^ drives tumor progression and invasion of distal organs, a process similar to metastasis in humans.^38^ However, it is not known whether this is specific to M6 and its role during tricellular SJ assembly, or whether this is related to SJ function, a question we address in the present study.

*Drosophila* is a widely used *in vivo* model in cancer biology for its versatile genetic toolbox, short generation time and cancer-related genes conserved in humans. Studies with *Drosophila* have characterized Ras-mediated tumorigenesis at the molecular and tissue level, identified tissue-intrinsic and -extrinsic cooperative pathways during tumor progression, and developed personalized pharmacological treatments for patients with cancer.^12^^,^^39^^,^^40^^,^^41^^,^^42^^,^^43^^,^^44^ Here, we used the larval eye-antenna imaginal disc of *Drosophila* to study the role of SJ in epithelia homeostasis and tumorigenesis. We found that SJ ensures epithelia homeostasis by preventing apoptosis in epithelial cells and that the disruption of SJ integrity in conjunction with *Ras*^*V12*^ promotes neoplastic transformation and delamination. We found *Ras*^*V12*^-cells depleted in SJ delaminate apically from squamous epithelia and basally from pseudostratified epithelia, where they also form tissue folds with different junctional remodeling depending on cell identity. We adapted a 2D-agent model^45^^,^^46^ to the eye disc and investigated the respective contribution of tissue architecture, cell adhesion, and matrix stiffness during delamination. Our results highlight that the directionality of delamination is governed by tissue-intrinsic properties, namely tissue cytoarchitecture, and preferential adhesion between mutant cells with each other and with wild-type cells. Our results reveal a non-occluding function of SJ in preserving epithelial homeostasis.

## Results

**Figure 1:**
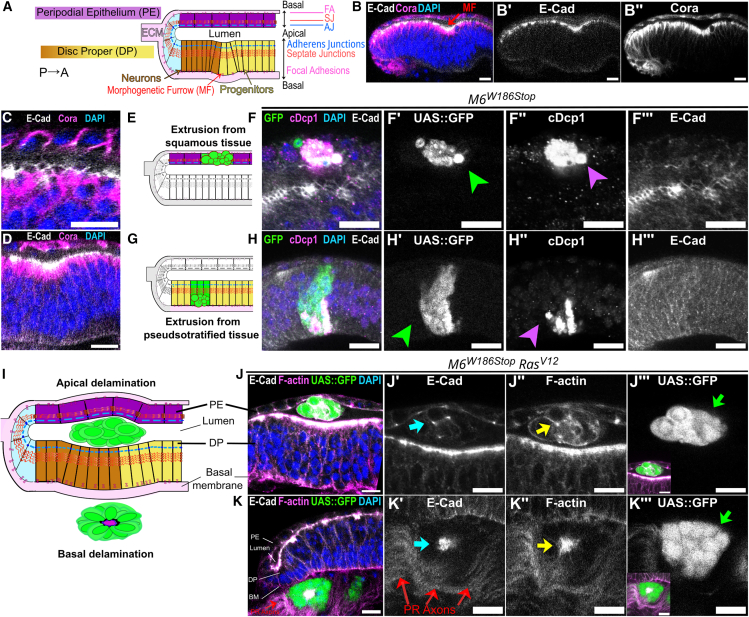
Loss of septate junction integrity in *Ras*^*V12*^ expressing cells promotes delamination in the *Drosophila* eye disc (A, E, G, and I) Schemes of orthogonal sections along the dorsoventral axis of the *Drosophila* eye disc (A), apical and basal extrusion (C and E) and apical and basal delamination (G). In this and following panels, the posterior-to-anterior axis is left-to-right, the peripodial epithelium (PE) in violet, undifferentiated cells in the Disc Proper (DP) in yellow, differentiated cells in the DP in brown, adherens junctions (AJ) in blue, septate junctions (SJ) in red, focal adhesions (FA) in magenta, the extracellular matrix (ECM) in light magenta. Green cells represent clones generated using the eyMARCM system. (B–D) Wild-type eye disc stained for E-cad, Coracle, and DAPI during neurogenesis, where the differentiation forefront (morphogenetic furrow, MF) can be seen (B, red arrow) and the squamous peripodial epithelium (C) and pseudostratified disc proper (E) are shown as insets. (F and H) Eye discs stained for E-cad and cleaved-*Drosophila* caspase-1 (cDcp1). M6 clones (*M6*^*W186Stop*^, green arrowheads) in the squamous epithelium (F–F‴) or in the undifferentiated pseudostratified epithelium (H–H‴) contain pyknotic nuclei (orange arrowheads) and are caspase-activated (violet arrowheads). (J–J‴) *M6*^*W186Stop*^-*Ras*^*V12*^ clones (green) apically delaminate in the lumen with E-cad and F-actin located along the cell membranes. (K–K‴) *M6*^*W186Stop*^-*Ras*^*V12*^ clones (green) basally delaminate as rosettes, with E-cad and F-actin in the central domain. Photoreceptor axons (PR axons, red arrows) are seen in the posterior part of the disc. Scale bars represent 10 μm. Each image is a single confocal optical section. Posterior left, dorsal up. See Figure S1. See supplemental information for complete genotypes and sample sizes.

To study the contribution of SJ to collective delamination in conjunction with *Ras*^*V12*^, we used the *Drosophila* eye disc composed of a squamous peripodial epithelium (PE) and a pseudostratified disc proper (DP) separated by a lumen and facing each other through their apical domains (Figures 1A and 1B). Neurogenesis in the pseudostratified epithelium starts in late larval development (L3) and progresses in a posterior-to-anterior fashion.^47^ The differentiation forefront, observed as a transient indentation in the tissue, is termed morphogenetic furrow (MF) (Figures 1A–1B″). Equipotent cells anterior to the MF are progressively patterned in an ommatidia lattice posterior to the MF.^48^ In the squamous (Figure 1C) and pseudostratified epithelium (Figure 1D), AJ main component E-cadherin (E-cad) is apical to bicellular (Coracle) and tricellular (Anakonda and Gliotactin) core-components of SJ (Figures S1A–S1B″). We used the Mosaic Analysis with Repressible Clone Marker (MARCM) system^49^^,^^50^ and generated heterozygote eye discs containing wild-type and SJ-homozygote mutant cells. The *ey-*Flipase, expressed under the control of the *eyeless* promoter, induces tissue-specific FRT-site specific mitotic recombination that, combined with the UAS/Gal4/Gal80 system, generates SJ-mutant cells expressing UAS-driven transgenes such as the GFP clone marker and, whenever present, *Ras*^*V12*^.

### Loss of septate junction integrity promotes cell death and extrusion

M6 clones, homozygote for a loss-of-function null allele (*M6*^*W186Stop*^), expressing the GFP-clone marker (Figures 1E–1H‴) were generated in the squamous (Figures 1E–1F‴) and the pseudostratified epithelium, anterior (Figures 1G–1H‴) and posterior (Figures S1C–S1D‴) to the MF. M6 clones exhibited pyknotic nuclei (Figures 1F–1H, and S1D, green arrowheads), a sign of cell death. Sample size for this and the following experiments is detailed in Table 1. Cell extrusion eliminates caspase-activated cells from the epithelium during normal development and tissue surveillance.^51^ To determine whether M6 clones underwent apoptosis, we studied the marker of early caspase-activation, cleaved-*Drosophila* caspase-1 (cDcp1). Developmentally programmed cell death occurs at the pupal stage in the pseudostratified epithelium, posterior to the MF (Wolff & Ready, 1991), hence we assessed cell survival during early larval development, anterior to the MF. In contrast to the control situation, we found that over half of M6 clones in the squamous epithelium and anterior to the MF in the pseudostratified epithelium were positive for cDcp1 in L2 and early L3 stages (Figures 1F and 1H, violet arrowheads). To determine whether caspase-activation was specific to M6-depletion, we studied the consequence of the loss of bicellular (*nrv2*^*K13315*^, a hypomorphic allele^52^) and tricellular (*aka*^*L200*^, a null allele^53^) SJ core-components. In both situations, pyknotic nuclei were observed in mutant cells in the squamous (Figures S1E–S1G″) and pseudostratified (Figures S1H–S1J″) epithelium. These results show that the loss of bi- or tri-cellular SJ core-components triggers the apoptosis of some of the mutant cells and that dying cells extrude without causing any detectable tissue deformation.

**Table 1:** Sample size from each Figure panel

Figure	Location of clones	Number of events (n)	Number of eye discs (N)	Independent experiments
Figures 1D–1F	Squamous epithelium	47	26	19
	Pseudostratified epithelium (undifferentiated)	132	45	20
Figure 1H	Lumen	40	31	18
Figure 1I	Basal to pseudostratified epithelium	57	34	20
Figure S1B	Pseudostratified epithelium (differentiated)	26	15	7
Figures 2B and 2C	Pseudostratified epithelium (undifferentiated)			
	*M6*^*W186Stop*^*Ras*^*V12*^ clones	3	3	3
	*aka*^*L200*^*Ras*^*V12*^ clones	4	3	3
Figures 2E and 2F	Pseudostratified epithelium (undifferentiated)			
	*M6*^*W186Stop*^*Ras*^*V12*^ clones	269	85	45
	*aka*^*L200*^*Ras*^*V12*^ clones	146	46	24
	*nrv2*^*K13315*^*Ras*^*V12*^ clones	22	10	5
Figures 2H and 2I	Pseudostratified epithelium (undifferentiated)			
	*M6*^*W186Stop*^*Ras*^*V12*^ clones	114	46	30
	*aka*^*L200*^*Ras*^*V12*^ clones	102	31	22
	*gli*^*DV3*^*Ras*^*V12*^ clones	8	3	1
	*nrv2*^*K13315*^*Ras*^*V12*^ clones	9	6	4
Figures S2B and S2C	Squamous epithelium	24	17	6
	Pseudostratified epithelium (undifferentiated)	27	12	4
Figures S2E–S2G	Squamous epithelium			
	*aka*^*L200*^*Ras*^*V12*^ clones	18	13	9
	*nrv2*^*K13315*^*Ras*^*V12*^ clones	6	4	4
Figures S2F–S3H	Pseudostratified epithelium			
	*aka*^*L200*^*Ras*^*V12*^ clones	31	22	17
	*gli*^*DV3*^*Ras*^*V12*^ clones	3	2	1
	*nrv2*^*K13315*^*Ras*^*V12*^ clones	21	12	9
Figures 3B and 3C	Pseudostratified epithelium (differentiated)			
	*M6*^*W186Stop*^*Ras*^*V12*^ clones	70	21	15
	*aka*^*L200*^*Ras*^*V12*^ clones	66	15	8
	*gli*^*DV3*^*Ras*^*V12*^ clones	27	7	3
	*nrv2*^*K13315*^*Ras*^*V12*^ clones	17	4	2
Figures 3E and 3F	Pseudostratified epithelium (differentiated)			
	*M6*^*W186Stop*^*Ras*^*V12*^ clones	127	45	28
	*aka*^*L200*^*Ras*^*V12*^ clones	81	28	17
	*gli*^*DV3*^*Ras*^*V12*^ clones	22	12	4
	*nrv2*^*K13315*^*Ras*^*V12*^ clones	40	10	5
Figure S3B	Pseudostratified epithelium (undifferentiated)	9	5	3
Figure S3D	Pseudostratified epithelium (undifferentiated)	2	2	2
Figure S3F	Pseudostratified epithelium (differentiated)	28	10	3
Figure S3H	Pseudostratified epithelium (differentiated)	48	19	6
Figure S3J	Basal to pseudostratified epithelium	9	6	3
Figure S3K	Basal to pseudostratified epithelium			
	*M6*^*W186Stop*^*Ras*^*V12*^ clones	2	2	1
	*aka*^*L200*^*Ras*^*V12*^ clones	3	2	1
Figure 4B	Pseudostratified epithelium (undifferentiated)	39	15	7
Figure 4D	Pseudostratified epithelium (undifferentiated)	4	3	2
Figure 4F	Pseudostratified epithelium (differentiated)			
	*M6*^*W186Stop*^*Ras*^*V12*^ clones	21	10	4
	*aka*^*L200*^*Ras*^*V12*^ clones	38	16	7
Figure 4H	Pseudostratified epithelium (differentiated)			
	*M6*^*W186Stop*^*Ras*^*V12*^ clones	48	24	9
	*aka*^*L200*^*Ras*^*V12*^ clones	87	34	10
Figure 4J	Basal to pseudostratified epithelium	6	5	4
Figures 5B and 5C	Squamous epithelium			
	*M6*^*W186Stop*^*Ras*^*V12*^ clones	160	81	63
	*aka*^*L200*^*Ras*^*V12*^ clones	110	68	44
	*gli*^*DV3*^*Ras*^*V12*^ clones	3	3	2
	*nrv2*^*K13315*^*Ras*^*V12*^ clones	16	11	7
Figures 5E and 5F	Squamous epithelium			
	*M6*^*W186Stop*^*Ras*^*V12*^ clones	79	45	25
	*aka*^*L200*^*Ras*^*V12*^ clones	54	34	19
	*gli*^*DV3*^*Ras*^*V12*^ clones	7	5	2
	*nrv2*^*K13315*^*Ras*^*V12*^ clones	6	6	5
Figures 5H and 5I	Squamous epithelium			
	*M6*^*W186Stop*^*Ras*^*V12*^ clones	16	14	12
	*aka*^*L200*^*Ras*^*V12*^ clones	18	13	9
	*nrv2*^*K13315*^*Ras*^*V12*^ clones	6	4	4
Figure 7B	Squamous epithelium	28	12	11
Figure 7C	Squamous epithelium	25	15	6
Figures 7E and 7F	Squamous epithelium	36	18	13

### Loss of septate junction components in conjunction with *Ras*^*V12*^ promotes collective delamination

The loss of M6 in *Ras*^*V12*^-cells promotes delamination and invasiveness.^38^ To determine whether this is exclusive to M6, or a consequence of the loss of SJ integrity, we generated clones of bi- or tri-cellular SJ core-components expressing *Ras*^*V12*^. *M6*^−/−^*-Ras*^*V12*^ clones were negative for cDcp1 (Figures S2A–S2C‴), hence refractory to apoptosis, as expected from *Ras*^*V12*^ expression. *M6*^−/−^*-Ras*^*V12*^ clones underwent apical and basal collective delamination and appeared detached in the lumen or basal to the eye disc, respectively (Figures 1J and 1K). Together, the results indicate that *M6*^−/−^*-Ras*^*V12*^-cells delaminate collectively and live out of the epithelium. Similarly, *aka*^−/−^*-Ras*^*V12*^ clones and *nrv2*^−/−^*-Ras*^*V12*^ clones underwent apical (Figures S2E, S2E′, S2F, and S2F′) and basal (Figures S2G–S2G″ and S2H–S2H″) collective delamination. Junctional remodeling and cell organization differed between apical and basal delamination. Apically delaminated *SJ*^−/−^*-Ras*^*V12*^ clones appeared clustered in the lumen, and E-cad localized along the lateral plasma membrane (Figures 1J, S2B′, S2E″, and S2F″; Video S1). Conversely, basally delaminated *SJ*^−/−^*-Ras*^*V12*^ clones organized radially forming rosettes and E-cad was restricted to the central domain (Figures 1K, S2C′, S2G″, and S2H″; Video S2). Together, these findings indicate that perturbing SJ integrity in *Ras*^*V12*^-cells results in the collective delamination of living cells. These striking differences prompted us to question whether junctional remodeling during apical and basal delamination differed, a question we addressed next.

Video S1. Apically delaminated clones detach from the tissue and appear in the lumen, related to Figure 1

Video S2. Basally delaminated clones detached from the tissue locate basal to the eye disc, related to Figure 1

### Junctional remodeling during tissue folding differs depending on cell identity

**Figure 2:**
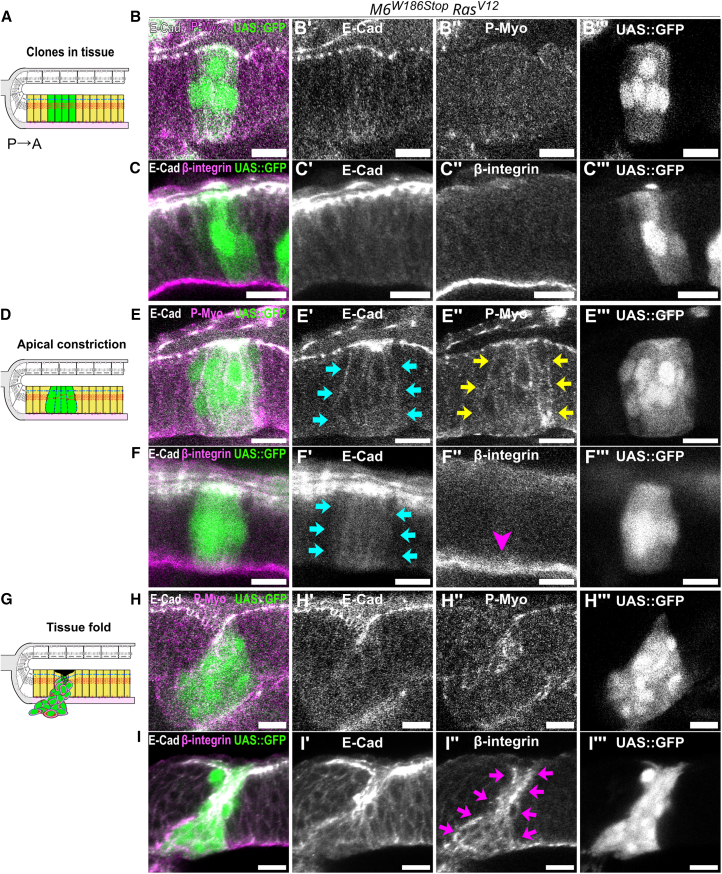
Basolateral localization of E-cad and β-integrin in SJ-depleted *Ras*^*V12*^ clones, anterior to the MF forming tissue folds (A, D, and G) Scheme of orthogonal sections along the dorsoventral axis of the *Drosophila* eye disc with SJ-depleted *Ras*^*V12*^*clones* anterior to the MF, forming tissue folds. (B, C, E, F, H, and I) Eye discs stained for E-cad (gray) and P-Myo (B, E, and H, magenta) or β-integrin (C, F, I, magenta) containing SJ-depleted tumors (green), anterior to the MF. (B–B‴ and C–C‴) *SJ*^−/−^*-Ras*^*V12*^ clones (green) resemble wild-type neighbors in terms of adherens junctions, focal adhesions, and cytoskeleton remodeling. (E–E‴ and F–F‴) Apical constriction in *SJ*^−/−^*-Ras*^*V12*^ clones (green) is concomitant to basolateral spreading of E-cad (blue arrows) and P-Myo (green arrows), while β-integrin remains basal (magenta arrowhead) and anchoring to the matrix is preserved. (H–H‴ and I–I‴) SJ-depleted *Ras*^*V12*^ clones (green) form tissue folds with basolateral spreading of E-cad, P-Myo, and β-integrin (magenta arrows). Scale bars represent 10 μm. Each image is a single confocal optical section. Posterior left, dorsal up. See supplemental information for exact genotypes and sample sizes.

**Figure 3:**
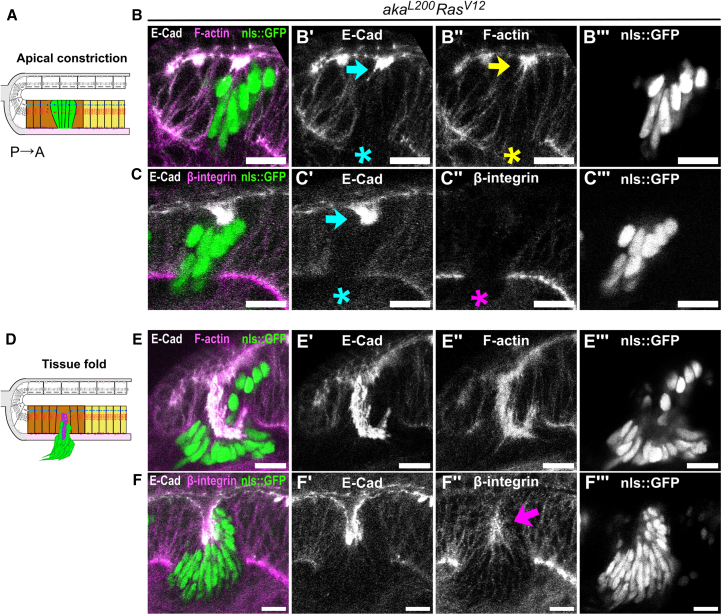
β-integrin localizes apically in SJ-depleted *Ras*^*V12*^ clones, forming tissue folds posterior to the MF (A and D) Scheme of orthogonal section along the dorsoventral axis of the *Drosophila* eye disc with SJ-depleted *Ras*^*V12*^ clones forming tissue folds posterior to the MF. (B, C, E, and F) Eye discs stained for E-cad (gray), F-actin (B, E, H magenta) or β-integrin (C, F, magenta). (B–B‴ and C–C‴) *SJ*^−/−^*-Ras*^*V12*^ clones (*aka*^*L200*^*-Ras*^*V12*^, green) in the differentiated region of the pseudostratified epithelium constrict apically (blue and yellow arrows) and lose basal E-cad (blue asterisk), β-integrin (magenta asterisk), and actin stress fibers (yellow asterisk), suggesting a loss of basal anchoring to the matrix. (E–E‴ and F–F‴) *SJ*^−/−^*-Ras*^*V12*^ clones invaginate and β-integrin is apically enriched (magenta arrow), suggesting preferential adhesion between mutant cells. Scale bars represent 10 μm. Each image is a single confocal optical section. Posterior left, dorsal up. See supplemental information for exact genotypes and sample sizes.

To characterize the sequence of events leading to apical and basal delamination, we staged eye discs at different time points during larval development. We characterized the changes in localization of cell adhesion and cytoskeleton markers during delamination (see STAR Methods). Cell-cell and cell-matrix junctional remodeling were monitored using E-cad and β-integrin, respectively. Cytoskeleton remodeling was monitored using anti-phosphorylated regulatory light chain of non-muscle type II myosin (P-Myo) as a proxy for active MyoII, and phalloidin to study filamentous actin (F-actin). From late L2 until L3 stages, *SJ*^−/−^*-Ras*^*V12*^ clones in the undifferentiated region of the pseudostratified epithelium, anterior to the MF, showed a gradation in the phenotype associated with clone size. Small clones of less than 5 cells were wild-type like (Figures 2A–2C‴). Intermediate clones of 6–10 cells constricted apically, displayed basolateral spreading of E-cad and P-Myo. In these clones, β-integrin localized basally (Figures 2D–2F‴), suggesting they remain anchored to the matrix. Large clones of over 20 cells were apically constricted and formed tissue folds with a basolateral spreading of E-cad and P-Myo. In these clones, β-integrin localized at the lateral and apical surfaces instead of being restricted basally (Figures 2G–2I‴). During L3, *SJ*^−/−^*-Ras*^*V12*^ clones in the differentiated region of the pseudostratified epithelium, posterior to the MF, displayed a different gradation in phenotype. Intermediate clones of 6–10 cells underwent apical constriction and lost E-cad basally. These clones also lost β-integrin and F-actin stress fibers basally, suggesting a detachment from the basal matrix (Figures 3A–3C‴). Large clones of more than 20 cells constricted apically and formed tissue folds basally depleted in E-cad, β-integrin, and F-actin, with β-integrin localized apically (Figures 3D–3F‴). These findings show that *SJ*^−/−^*-Ras*^*V12*^ clones in the pseudostratified epithelium disrupt tissue architecture and trigger collective cell movements with a basal directionality. Finally, *SJ*^−/−^*-Ras*^*V12*^ clones in the pseudostratified epithelium were found basally delaminated in the form of rosettes. Whether rosettes originate from *SJ*^−/−^*-Ras*^*V12*^ clones located anterior or posterior to the MF was next addressed.

### Septate junction-depleted *Ras*^*V12*^ clones delaminate basally and form rosettes containing differentiated cells

**Figure 4:**
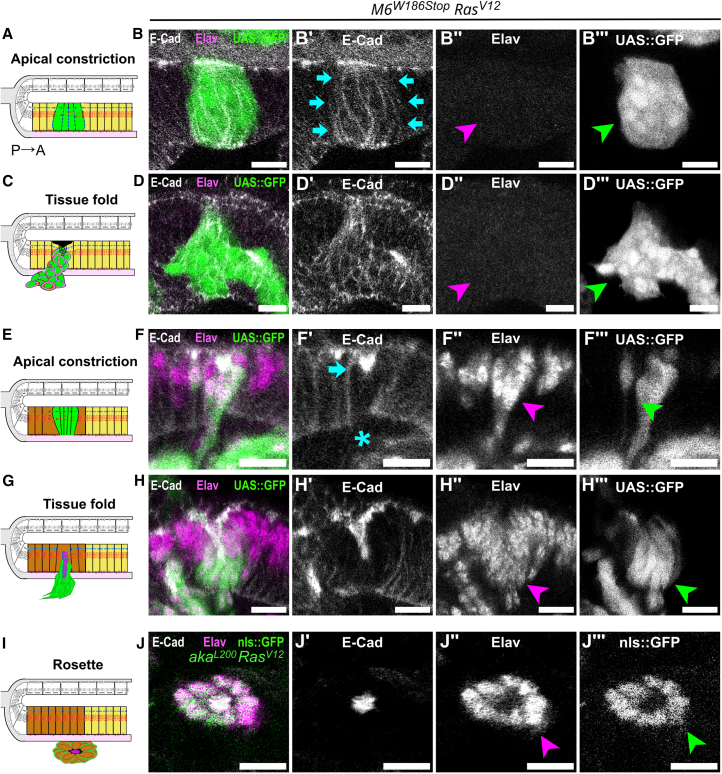
SJ-depleted *Ras*^*V12*^ clones delaminate basally to form rosettes containing polarized, differentiated cells (A, C, E, G, and I) Schemes of orthogonal sections along the dorsoventral axis of the *Drosophila* eye disc with SJ-depleted-*Ras*^*V12*^ clones (green) undergoing collective multicellular rearrangements in the pseudostratified epithelium, anterior (yellow) or posterior (brown) of the MF. (B, D, F, H, and J) Heterozygous eyes stained for E-cad (gray) and Elav (magenta). (B–B‴ and D–D‴) SJ-depleted *Ras*^*V12*^ clones (*M6*^*W186Stop*^^*-*^*Ras*^*V12*^, green) anterior to the MF are negative for the Elav marker (magenta arrowhead), hence undifferentiated, when undergoing apical constriction and basolateral E-cad delocalization (B, blue arrows) and invagination during tissue folding (D). (F–F‴ and H–H‴) SJ-depleted *Ras*^*V12*^ clones (*M6*^*W186Stop*^*-Ras*^*V12*^, green) posterior to the MF express the Elav marker (F″, H″, magenta arrowheads), hence are differentiated, during apical constriction (F, blue arrow), concomitant to the basal loss of E-cad (F, blue asterisk) and form tissue folds (H). (J–J‴) SJ-depleted *Ras*^*V12*^ clones (*aka*^*L200*^*-Ras*^*V12*^ green) delaminate basally and form rosettes that exclusively contain Elav-expressing cells (J″, magenta arrowhead). Scale bars represent 10 μm. Each image is a single confocal optical section. Posterior left, dorsal up. See also Figure S3. See supplemental information for exact genotypes and sample sizes.

To decipher whether rosettes originate from *SJ*^−/−^*-Ras*^*V12*^ clones located anterior or posterior to the MF, we next studied cell identity using the marker of early neural differentiation Elav (Robinow & White, 1991). Anterior to the MF, apically constricted intermediate-size clones (Figures 4A–4B‴) and large clones forming tissue folds (Figures 4C–4D‴) were Elav-negative, hence undifferentiated. Posterior to the MF, small clones undergoing apical constriction (Figures 4E–4F‴) and large clones forming tissue folds (Figures 4G–H‴) were Elav-positive, hence differentiated. Rosettes were systematically found basal to the differentiated region of the pseudostratified epithelium and were consistently Elav-positive (Figures 4I–4J‴). *SJ*^−/−^*-Ras*^*V12*^ clones formed anterior to the MF exhibited a partial delocalization to the basolateral pole of E-Cad and atypical protein kinase C (aPKC) from the apical Par complex^54^ (Figures S3A–S3D‴). In contrast, *SJ*^−/−^*-Ras*^*V12*^ clones posterior to the MF exhibited a correct localization of E-Cad and aPKC, restricted to the apical domain, suggesting that cell polarity is preserved. (Figures S3E–S3H‴). In rosettes, E-cad and aPKC were restricted to the center (Figures S3I–S3J‴) along with β-integrin (Figures S3K–S3K‴). These results suggest that *SJ*^−/−^*-Ras*^*V12*^ clones in the pseudostratified epithelium, posterior to the MF, delaminate basally and form rosettes.

### Septate junction-depleted *Ras*^*V12*^ clones delaminate apically from the squamous epithelium

**Figure 5:**
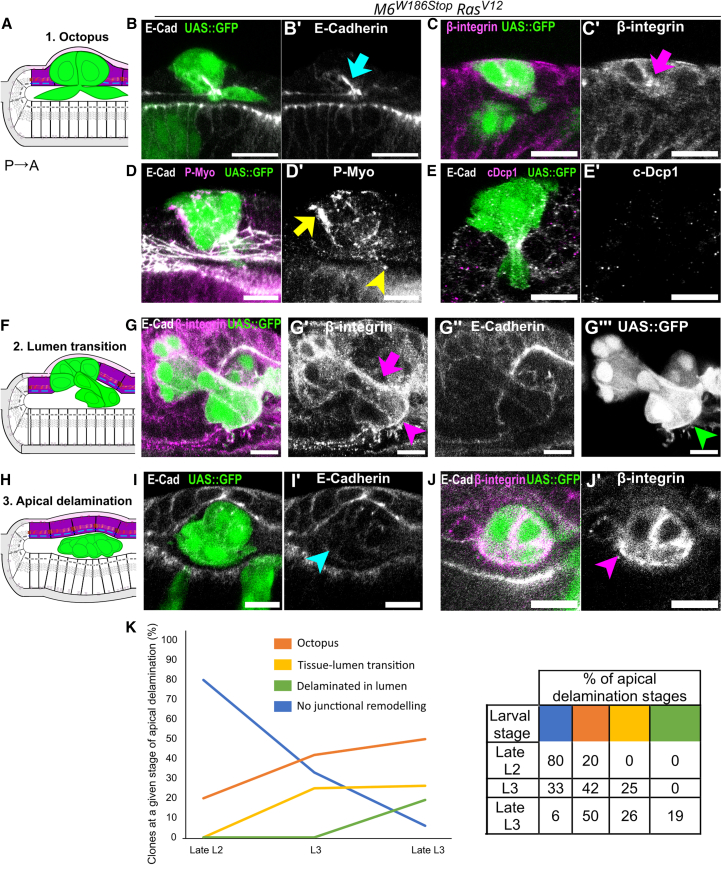
SJ-depleted *Ras*^*V12*^ clones undergoing apical delamination constrict apically, translocate into the lumen and detach from the squamous epithelium (A, F, and H) Schemes of orthogonal sections along the dorsoventral axis of the *Drosophila* eye disc in the squamous epithelium (purple) undergoing apical delamination. (B, C, E, G, I, and J) Heterozygous eyes stained for E-cad (gray), Cora (B, E, H, magenta) or β-integrin (C, G, J, magenta). (B–E′) SJ-depleted *Ras*^*V12*^ clones (*M6*^*W186Stop*^*-Ras*^*V12*^, green) in the squamous epithelium at the octopus stage constrict apically, acquire a dome shape, exhibit the basolateral enrichment of E-cad (B′, blue arrow) and β-integrin (C′, magenta arrow). Also at this stage, Myo is activated at the clone boundary (D′, yellow arrow) and at the tip of lumen protrusions (D′, yellow arrowhead) as clones delaminate alive out of the epithelium, as shown by the absence of *Drosophila-*caspase 1 activation (E and E′). (G–G‴) SJ-depleted *Ras*^*V12*^ clones (*M6*^*W186Stop*^*-Ras*^*V12*^, green) undergoing apical delamination are enriched in β-integrin (G′, magenta arrow) as well as cells that have already translocated into the lumen (G′, magenta arrowhead; G″, green arrowhead). (I–J′) Apically delaminated SJ-depleted *Ras*^*V12*^ clones (*M6*^*W186Stop*^-*Ras*^*V12*^, green) are detached from the squamous epithelium (I′, blue arrowhead) and enriched in β-integrin (J′, magenta arrowhead). (K) The percentage of clones at different stages of apical delamination evolved over time. Clones with no junctional remodeling (blue) appeared later in development, whereas clones undergoing apical constriction (orange) and translocating in the lumen (yellow) were more frequent later in development. Clones having completed apical delamination (green) were absent in early L2 stages and appeared in the lumen toward the end of L3 stage. See STAR Methods. Scale bars represent 10 μm. Each image is a single confocal optical section. Posterior left, dorsal up. See supplemental information for exact genotypes and sample sizes.

Our analyses on fixed specimens at different time-points during larval development revealed that *SJ*^−/−^*-Ras*^*V12*^ clones in the squamous epithelium delaminate apically. In the late L2 stage, *M6*^−/−^*-Ras*^*V12*^ clones showcased no junctional remodeling. In L3 stage, clones with an average size of 4–8 cells constricted apically, acquired a dome-like shape, and emitted lumen protrusions. Clones in this stage, termed “octopus” due to the morphology, were laterally enriched in E-cad and β-integrin (Figures 5A–5C′). At this stage, P-Myo was present at the tip of lumen protrusions and at the boundary between wild-type and mutant cells (Figures 5D and 5D′). The latter was reminiscent of the supracellular cable that forms around dying cells during cell extrusion in the pupal abdomen^10^ and notum.^11^ However, unlike extruding cells which are caspase-activated, we observed no caspase activation during apical delamination (Figures 5E and 5E′), suggesting this process occurs independently of apoptosis. In L3, clone size increased to 10–15 cells and mutant cells translocated into the lumen. Cells remained cohesive and β-integrin was enriched in cells embedded in the layer, in cells translocating in the lumen, and in cells detached from the epithelium (Figures 5F–5G‴). During late L3, cluster size increased to 15–23 cells and mutant cells appeared detached in the lumen (Figures 5H–5I′), enriched in β-integrin (Figures 5J and 5J′). The percentage of *M6*^−/−^*-Ras*^*V12*^ clones at any given stage of apical delamination evolved over time (Figure 5K, see Table S1). Initially high, the percentage of *M6*^−/−^*-Ras*^*V12*^ clones showcasing no junctional remodeling decreased over time, while clones at the octopus stage or translocating into the lumen appeared more frequently at later developmental stages. Clones having completed apical delamination from the squamous epithelium only appeared at later stages of larval development. The distinct junctional remodeling across apical and basal delamination events prompted us to query how the directionality of delamination is governed.

### Events of epithelia destabilization *in silico* recapitulate *in vivo* outcomes

**Figure 6:**
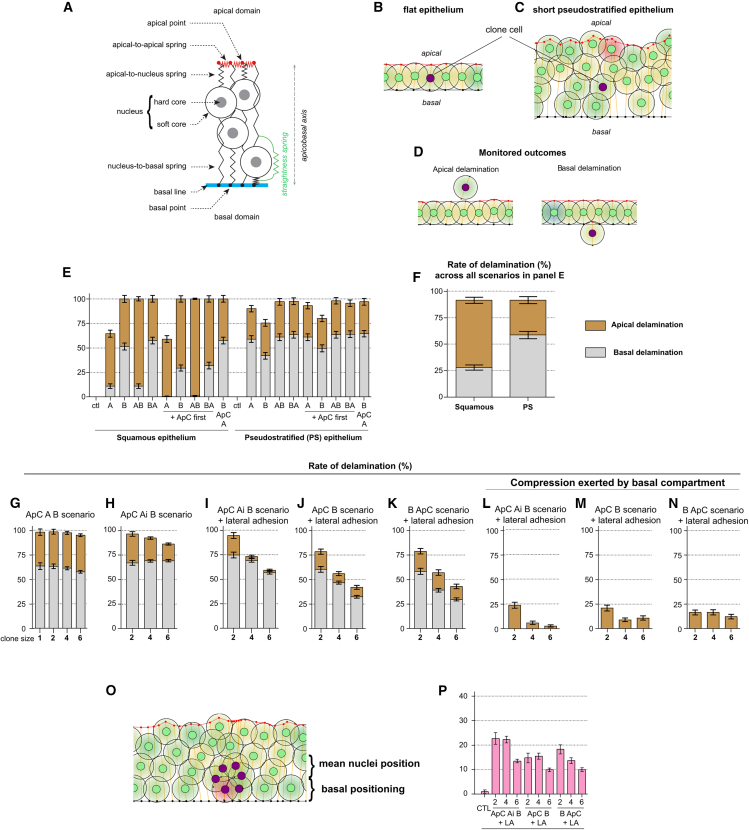
Outcomes of epithelia destabilization events in squamous and pseudostratified tissues differ *in silico* (A) Diagram representing the simulated cells and the various adjustable springs controlling their viscoelastic properties. Cells are seeded on a non-deformable basal line above a matrix-like compartment acting as a physical barrier whose resistance strength is tunable (see STAR Methods). (B and C) Examples of modeling outcomes showing the epithelium in squamous or short pseudostratified configuration. Wild-type cells have green nuclei, clone cells have magenta nuclei. (D) Examples of apical and basal delamination upon epithelia destabilization. (E) Mean rates of delamination with standard error of the mean for each tested scenario in single cells. (F) Global rate of delamination for all simulations shown in panel E. (G–N) Mean rates of delamination with standard error of the mean for all indicated scenarios tested in clones of various sizes. Each simulation was performed 200 times. (L–N) Mean rates of delamination with standard error of the mean for all indicated scenarios tested in clones of various sizes in the presence of a non-permissive basal compartment. Each simulation was performed 200 times. (O) Example of a clone (magenta) that underwent the ApC B and lateral adhesion scenario, note that only some of the clone cells are considered basally positioned. (P) Rates of basal positioning for each tested scenario. A, loss of apical adhesion between clone cells and between clone and wild-type cells; Ai, loss of apical adhesion only at the interface between clone cells and wild-type cells; ApC, apical constriction; B, loss of basal adhesion; PS, pseudostratified.

To assess the relative contribution of cell-cell and cell-ECM adhesion remodeling in controlling the apical versus basal directionality of delamination, we performed computational analyses. We adjusted a recently developed 2D agent-based model of epithelial dynamics^45^^,^^46^ (Figure 6A, see STAR Methods, see Table S2) to the eye disc architecture, obtaining a flat monolayer (Figure 6B; Video S3) and a cuboidal tissue (Figure 6C; Video S3), which biologically can be interpreted as a squamous and pseudostratified epithelium respectively, referred as such here after. We first implemented three parameters identified *in vivo* during delamination, namely apical constriction (event ***ApC***), the loss of apical cell-cell adhesion (event ***A***), and the loss of adhesion to the basal matrix (event ***B***). We implemented these events in clone cells and monitored the rate of apical and basal delamination (Figure 6D). We tested several scenarios and simulated these events occurring in a single cell, first independently and then sequentially in various orders (Figure 6E). All events were progressively implemented over 20 h, and epithelia were left to develop for an extra 30h after the last event so that the consequences of the events could be monitored.^46^ ***ApC*** alone did not trigger delamination but led to the basal displacement of the cell nucleus in the pseudostratified epithelium (Figure S4). In the short pseudostratified epithelium, basal delamination was systematically the main outcome regardless of the order of epithelia destabilization events (Figures 6E and 6F). In the squamous epithelium, most scenarios preferentially led to apical delamination (Figure 6E), unless ***B*** occurred first (Figure 6E), in which case the directionality of delamination was randomized. *SJ*^−/−^*-Ras*^*V12*^ clones delaminate collectively *in vivo*; therefore, we next studied whether clone size affected the directionality of delamination.

Video S3. Computational modelling of the eye-antennal imaginal disc using the EMT-simulator, related to Figure 6

### The architecture of the pseudostratified epithelium favors the basal directionality regardless of junctional remodeling, clone size, and matrix resistance

First, we focused on the pseudostratified epithelium, where the directionality of delamination characterized *in vivo* was preserved *in silico* in single cells, regardless of the order of epithelia destabilization events. In 3D, clone size would be triple the corresponding size in the 2D-agent model. Therefore, to query biologically relevant, scenarios, we restrained clone size to our *in vivo* data. The ***ApC A B*** scenario comprises all events observed *in vivo* during basal delamination. We found that increasing clone size in this epithelium had a marginal effect on the directionality of delamination, with the majority of clones delaminating basally (Figure 6G; Video S4). Surprisingly, in these conditions, we observed clone splitting, a phenomenon in which cells from the same clone delaminated on different sides of the epithelium (Video S4). Clone splitting was never observed *in vivo*, raising the question as to whether adhesion between clone cells is needed during collective delamination.

**Figure 7:**
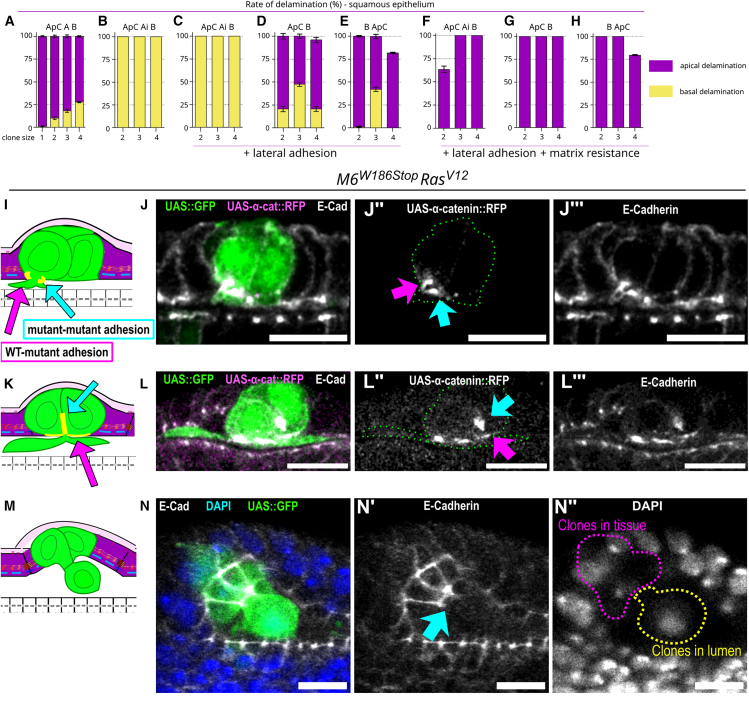
Preferential adhesion between wild-type and mutant cells regulates the directionality of apical delamination (A–H) Mean rates of delamination with standard error of the mean for all indicated scenarios tested in clones of various sizes. Each simulation was performed 200 times. (L–N) Mean rates of delamination with standard error of the mean for all indicated scenarios tested in clones of various sizes in the presence of a non-permissive basal compartment. Each simulation was performed 200 times. A, loss of apical adhesion between clone cells and between clone and wild-type cells; Ai, loss of apical adhesion only at the interface between clone cells and wild-type cells; ApC, apical constriction; B, loss of basal adhesion; PS, pseudostratified. (I, K, and M) Schematic of an orthogonal section along the dorsoventral axis of the *Drosophila* eye disc of the squamous epithelium (purple). Mutant cells (green) apically constrict (I) into a dome-shaped octopus with apical protrusions (K) and progressively translocate into the lumen (M). (J–J″ and L–L‴) Heterozygous eye disc stained with anti-E-cad (gray) and expressing α-catenin::RFP (magenta) exclusively in SJ-depleted *Ras*^*V12*^ clones (*M6*^*W186Stop*^*-Ras*^*V12*^, green). During apical delamination, when SJ-depleted *Ras*^*V12*^ clones (*M6*^*W186Stop*^*-Ras*^*V12*^, green) start to emit lumen protrusions (J–J‴) and at the octopus stage (L–L‴), α-catenin::RFP (magenta) is found at the boundary between mutant cells (blue arrows) and between wild-type and mutant cells (magenta arrows). (N–N‴) During apical delamination, SJ-depleted *Ras*^*V12*^ clones (*M6*^*W186Stop*^*-Ras*^*V12*^, green) translocate into the lumen via an apical neck (E-cad, blue arrow). Dotted lines show the part of the clone where cells are embedded in the squamous epithelium (magenta) and the cell from the clone that has translocated into the lumen (yellow). Scale bars represent 10 μm. All images are single confocal optical sections with posterior to the left, anterior to the right. See supplemental information for exact genotypes and sample sizes.

Video S4. The outcome of epithelial destabilization is sensitive to clone size in squamous but not in pseudostratified epithelia, related to Figure 6 and Figure 7

To assess the contribution of cell adhesion to collective delamination, we introduced a new event called ***Ai,*** during which apical cell-cell adhesion is lost exclusively at the interface between clone and wild-type cells while adhesion between clone cells is preserved. When ***Ai*** occurs, the two control cells on either side of the clone form a new bond and “heal” the local wound (see STAR Methods). In the pseudostratified epithelium, maintaining adhesion between clone cells mildly reduced the rate of apical delamination but was not sufficient to prevent clone splitting (Figure 6H; Video S5). To further strengthen the adhesion between clone cells and mimic the maintenance of a strong lateral bond that prevents splitting, we implemented lateral adhesion by adding a maximum distance rule between clone cells. With the implementation of lateral adhesion on the simulations, upon loss of basal cell-matrix adhesion (***B***), clone cells cannot drift away from each other. In the pseudostratified epithelium, enforcing lateral adhesion suppressed clone splitting and made apical delamination a rare outcome (Figure 6I; Video S6). Reinforcing lateral adhesion between clone cells while preserving apical adhesion with wild-type cells (***Ai*** not occurring) was not sufficient to prevent apical delamination from occurring (Figures 6J and 6K). This suggests that other parameters may regulate the directionality of delamination.

Video S5. Moderate cell adhesion does not prevent clone splitting in the pseudostratified epithelium but reverses directionality in the squamous epithelium, related to Figure 6 and Figure 7

Video S6. Reinforced lateral adhesion between clones in pseudostratified epithelia suppresses clone splitting and favors basal delamination, related to Figure 6

Thus far, all simulations were performed in a permissive context. In this environment, cells, represented as nuclei, attach with a basal spring to the basal line and sit atop a basal compartment that acts as a substrate but not as a physical barrier. However, *in vivo*, the extracellular matrix in imaginal discs is known to act both as a substrate and a physical barrier.^55^^,^^56^ Therefore, we next studied how a non-permissive environment impacts the directionality of delamination. We simulated previous scenarios in a context where the basal compartment behaves like a substrate and a physical obstacle, forcing cells to experience a small degree of confinement (see STAR Methods). In the pseudostratified epithelium, a non-permissive basal compartment, which biologically can be interpreted as a basal matrix, prevented basal delamination from occurring but did not increase the rate of apical delamination (Figures 6L–6N; Video S7). We next studied whether the directionality of collective movement was affected in this tissue. To do so, we monitored the rate of basal positioning, which corresponds to the percentage of cells with their nuclei located more basal than the average nuclear position of wild-type cells (Figures 6O and 6P). Interestingly, all scenarios tested in a non-permissive environment led to the basal positioning of cells (Figure 6O). This indicates that despite preventing actual delamination due to the presence of a non-permissive basal compartment, which can be biologically interpreted as increasing the matrix resistance, the basal directionality is intrinsic to the pseudostratified epithelium following epithelia destabilization events.

Video S7. A non-permissive matrix prevents basal delamination in squamous epithelia but does not abrogate the intrinsic basal directionality of delamination upon epithelial destabilization in the pseudostratified layer, related to Figure 6

To further understand how tissue architecture, clone size, matrix resistance, and lateral adhesion regulate the directionality of delamination, we next queried these parameters in the squamous epithelium.

### The squamous epithelium is highly sensitive to junctional remodeling, clone size, and preferential adhesion regulation of the directionality of delamination

In the squamous epithelium, increasing clone size progressively increased the rate of basal delamination up to 25% of the cells (Figure 7A; Video S4). Strikingly, implementing ***Ai*** to remove cell adhesion between clone cells and wild-type neighbors completely reversed the directionality of delamination (Figure 7B; Video S5). Furthermore, reinforcing lateral adhesion between clone cells did not suffice to reverse the directionality of delamination if ***Ai*** was implemented (Figure 7C). To further assess the importance of preserving lateral adhesion during delamination, we simulated scenarios of ***ApC*** and *B* without ***Ai*** occurring and implemented lateral adhesion between wild-type and mutant cells. Preserving adhesion between wild-type and clone cells made apical delamination the main outcome (Figures 7D and 7E). Moreover, the presence of a non-permissive basal compartment abolished all events of basal delamination (Figures 7F–7H). This compressive environment was sufficient to promote apical delamination for all scenarios, including the ones that had an intrinsic bias toward basal delamination, such as the ***ApC Ai B*** scenario, in which lateral adhesion was lost between clones and wild-type neighbors (compare Figures 7C–7F).

Taken together, these numerical simulations predict that in the squamous epithelium, there are several possible outcomes depending on the type and order in which different events of epithelial destabilization occur. Among these outcomes, basal delamination might be prevented due to the presence of the matrix acting as a physical barrier, which biases delamination toward the apical side. During apical delamination, we observed that *in vivo* cells translocate collectively via an apical neck into the lumen. Hence, we next queried whether the preferential adhesion identified *in silico* to be required for the apical directionality was present during apical delamination *in vivo.* We studied the adhesion protein α-catenin, fluorescently tagged and expressed exclusively in mutant cells (UAS::α-catenin-RFP). We observed α-catenin to be present at the boundary between mutant cells and between mutant cells and wild-type neighbors early during the emission of lumen protrusions (Figures 7I–7J‴) and at the octopus stage (Figures 7K–7L‴). The site where lumen protrusions emanated resembled an apical neck (Figures S5A–S5B‴; Video S8). β-integrin was enriched in cells undergoing apical delamination and collectively translocating from the epithelium into the lumen (Figures S5C–S5E‴; Video S9). At the apical neck, nuclear deformation was present together with the cytoplasmic remnants from cells whose nuclei were already translocated in the lumen (Figures S5F–S5G‴; Video S10).

Video S8. Octopus stage during apical delamination, related to Figure 7

Video S9. Apical delamination from the squamous epithelium into the lumen occurs through an apical neck, related to Figure 7

Video S10. The apical neck presents the nucleus of a cell translocating into the lumen and the cytoplasm of cells with their nucleus already in the lumen, related to Figure S5 and Figure 7

## Discussion

We used the *Drosophila* eye disc and mathematical modeling to query the role of septate junctions in the homeostasis of squamous and pseudostratified epithelia during eye disc development and upon oncogenic *Ras*^*V12*^ expression. Our work reveals that in epithelial cells, the disruption of SJ integrity, following the loss of bicellular or tricellular SJ core-components, triggers caspase-activation and extrusion. Our *in silico* and *in vivo* results highlight how the disruption of SJ integrity in *Ras*^*V12*^-cells promotes basal collective delamination in the pseudostratified epithelium, regardless of the order of junctional remodeling. Conversely, *SJ*^−/−^*-Ras*^*V12*^ cells in the squamous epithelium exclusively undergo apical collective delamination. The directionality of delamination in this tissue is highly sensitive to the orchestration of junctional remodeling, and preferential adhesion between mutant and wild-type cells ensures collectiveness and completion of the process. Our results reveal a non-occluding function of SJ in epithelial homeostasis and tumorigenesis. Our findings reveal that the directionality of delamination during tumor progression is governed by tissue architecture and changes in cell adhesion.

The distinct outcomes of epithelial cells and *Ras*^*V12*^-cells following the disruption of SJ integrity may be due to differences in the relative fitness of each cell population. Cell competition is a tissue-surveillance mechanism that eliminates “less-fit” cells from the tissue in a context-dependent manner.^51^ The loss of Nrv2 disrupts SJ-integrity and mislocalizes Gli in the *Drosophila* notum,^27^ which triggers cell competition in the wing disc.^57^^,^^58^ The disruption of SJ-integrity also dysregulates the ESCRT/retromer system in the notum,^59^ which increases proteotoxic stress and activates cell competition in the wing disc.^60^ Cora contributes to cell survival^61^ and its depletion disrupts SJ-integrity and reduces proliferation,^62^ which can in turn trigger cell competition, as described in numerous organs.^63^ Ras overactivation makes cells refractory to apoptosis and can transform “less-fit” cells into “super-competitors,” ultimately promoting tumor development.^13^^,^^41^^,^^64^^,^^65^^,^^66^ To determine the mechanisms by which SJ-depletion triggers cell death but promotes neoplastic transformation of otherwise benign overgrowths, it would be informative to block cell death in SJ-mutant cells. This may help clarify whether making cells refractory to apoptosis bestows a fitness advantage sufficient to mask the “looser” status and can be achieved by expressing p35 in *SJ*^−/−^cells to then evaluate epithelia homeostasis.

### E-cadherin and β-integrin-based adhesion during epithelia folding

Tissue folds induced by *SJ*^−/−^*-Ras*^*V12*^ clones in the undifferentiated and differentiated region of the pseudostratified epithelium differ in terms of junctional remodeling at the apical, lateral, and basal domains, but preferential adhesion is present in both processes. It remains unclear whether the apical localization of β-integrin results from the absence of interaction with the ECM due to matrix degradation, or whether apically localized β-integrin somehow contributes to the cohesiveness of mutant cells at this location. In *Xenopus,* during the migration of neural crest cells, cadherins mediate adhesion with the matrix at basal focal adhesions.^67^ Interestingly, matrix-independent interplay between E-cad and β-integrin-mediated adhesion is present in cultured cells,^68^ and *in vivo* in embryonic^69^ and mature epithelia.^70^ Rap1, a small GTPase, and Canoe, an adherens junctions-cytoskeleton linker, regulate Cadherin- and Integrin-based adhesion modules during embryogenesis^71^^,^^72^^,^^73^^,^^74^ and eye development.^75^^,^^76^^,^^77^^,^^78^ In the eye disc, morphogens regulate the expression and stabilization of integrins, which in turn contribute to propagating the mechanochemical wave of differentiation.^79^ Hence, in addition to genetic programs driving cell shape changes, preferential cell adhesion and junctional remodeling appear to contribute to epithelia morphogenesis during tissue folding.

### Preferential adhesion and tissue architecture regulate the directionality of delamination

Our results confirm the synergy between M6 depletion and *Ras*^*V12*^ in promoting tumorigenesis.^38^ However, in the aforementioned study, it was proposed that apical delamination occurred in the pseudostratified epithelium across 190min, a phenomenon we never observed. It is unlikely we underscored any step during apical or basal delamination as we characterized junctional, cytoskeletal, cell identity and polarity markers in staged larvae with a 2h-time window, from 76h until 120h after egg laying (L2-L3). We also quantified nuclear size and noted that apically delaminated clones had a significantly bigger nuclear size than clones in the pseudostratified epithelium. The reason for this discrepancy remains unknown, but live imaging with clonal and junctional markers would help clarify this aspect.

Delamination is a critical first step in the epithelia to mesenchymal (EMT) transition^80^ and the directionality with which tumors delaminate impacts their malignancy.^81^ Similar to what we observe during apical delamination in *Drosophila*, tumors in vertebrates delaminate basally from the cortical domain farthest away from TJ, somewhat analogous to SJ. The hierarchy of junctional remodeling is critical during apical delamination in *Drosophila,* and E-cad remodeling is tightly controlled as single cells exit an epithelium.^82^^,^^83^^,^^84^ We find that preferential adhesion regulates the directionality of collective delamination in tumor progression and that E-cad and β-integrin-based adhesion are present and remodeled during this process. The bicellular and tricellular SJ core-components we found to synergize with Ras appear frequently mutated in carcinomas,^36^ strongly cementing the involvement of these junction components in the initiation of tumorigenesis. It would be informative to further assess the malignant capabilities of S*J*^−/−^*-Ras*^*V12*^ clones delaminating basally or apically. This can be achieved by generating clones exclusively in the squamous or the pseudostratified epithelium and assessing their ability to disseminate, invade, and colonize distal tissues. Furthermore, it would be informative to study the contribution of TJ components to cell delamination. Claudins are mutated in numerous cancers and basal delamination in vertebrates, the equivalent to apical delamination in invertebrates, is linked to metastasis and poor survival prognosis. An in-depth study on whether TJ core-components prevent delamination processes with different directionalities, and potentially distinct malignant capabilities, would be informative to elucidate non-occlusive functions of TJ, as described here for SJ, in tumor development.

### Limitations of the study

Based on cell polarity, identity, junctional and cytoskeleton markers, we report here that basally delaminated rosettes are formed by differentiated, polarized SJ-depleted *Ras*^*V12*^ clones originating in the posterior region of the pseudostratified epithelium. However, β-integrin is located in what seems to be the apical domain of the rosette, where E-cad, aPKC, and Crumbs are also found. This would argue in favor of a partial loss of polarity leading to β-integrin mislocalization during basal delamination, which is observed in SJ-depleted *Ras*^*V12*^ clones localized in the undifferentiated region of the pseudostratified epithelium. To conclude on the region within the pseudostratified epithelium where rosettes originate from, live imaging of basal delamination events in *ex vivo* explants of *Drosophila* eye discs would be required.

## Resource availability

### Lead contact

Resources and reagents are all available on request to the Lead Contact, Roland Le Borgne (roland.leborgne@univ-rennes1.fr).

### Materials availability

The study did not generate any unique reagents.

### Data and code availability

• This article includes all datasets generated or analyzed during this study. All data reported in this article will be shared by the lead contact upon request.
• Microscopy data reported in this article will be shared by the lead contact upon request.
• *In silico* simulations used a custom code, which is open source at https://github.com/SteffenPL/sEMTor.jl. *In silico* data were analyzed with Julia (v1.6), the code used for analysis is available open source at https://doi.org/10.24433/CO.7746744.v1.
• Any additional information required to reanalyze the data reported in this article is available from the lead contact upon request.

## Acknowledgments

We thank V. Auld, A. Bardin, S. Luschnig, T. Xu, and the Bloomington Stock Center and the National Institute of Genetics Fly Stock Center for providing fly lines. The monoclonal antibodies against Cora, E-cad, and β-Integrin were obtained from the Developmental Studies Hybridoma Bank, generated under the auspices of the National Institute of Child Health and Human Development, and maintained by the University of Iowa Department of Biological Sciences. We also thank S. Dutertre and X. Pinson from the Microscopy Rennes Imaging Center- BIOSIT. We are grateful to Chantal Roubinet and Caroline Dillard for their critical reading of the article.

This work was supported by grants awarded to MMO by Ligue Nationale Contre le Cancer (IP/SC-16639) and Association pour la Recherche contre le Cancer (ARCDOC42023020006318). SP is supported by the 10.13039/501100001691KAKENHI Grant-in-Aid for Early-Career Scientists (Grant number 24K16962). E.T. is supported by ANR-21-CE13-0028-02 and the ARC (ARCPJA22020060002084). RLB is supported by the French National Research Agency (ANR-20-CE13-0015) and ARC (PJA 20191209388). BIOSIT is a member of the national infrastructure France-BioImaging, supported by the 10.13039/501100001665ANR (ANR-10-INBS-04).

## Author contributions

R.L.B. and M.M.O. planned the project. M.M.O. did all the *in vivo* experiments and analyses. S.P. designed the *in silico* model adapted by E.T. and S.P. with help from discussions and *in vivo* data provided by M.M.O. and R.L.B. E.T. and S.P. performed all mathematical simulations and *in silico* analyses. All authors discussed the data. M.M.O. and R.L.B. wrote the article, and all authors gave comments on the article.

## Declaration of interests

The authors declare no competing interests.

## STAR★Methods

### Key resources table

**Table:** 

REAGENT or RESOURCE	SOURCE	IDENTIFIER
**Antibodies**		

Mouse anti- β-Integrin (1:250)	DSHB	Cat#CF.6G11; RRID:AB_528310
Mouse anti-Cora (1:200)	DSHB	Cat#C615.16; RRID:AB_1161644
Mouse anti-Elav (1:200)	DSHB	Cat#9F8A9; RRID:AB_528217
Rat anti-DE-cad (1:250)	DSHB	Cat#DCAD2; RRID:AB_528120
Mouse anti-Cleaved caspase-3 (1:1000)	Cell Signaling Technology	Cat#Asp175, RRID:AB_2341188
Mouse anti-Phospho-histone H3 (1:1000)	Cell Signaling Technology	Cat#Ser10 (6G3); RRID:AB_331748
Phospho-Myosin Light Chain 2 (1:250)	Cell Signaling Technology	Cat#Ser19; RRID:AB_2250969
Rabbit anti-Cleaved Drosophila Dcp-1 (1:100)	Cell Signaling Technology	Cat#Asp215; RRID:AB_2721060
Mouse anti-Gli (1:100)	Auld et al., 1995^85^	Cat#1F61D4 [48]
Alexa Fluor™ 647 Phalloidin (1:1000)	Thermo Fisher Scientific	Cat#A22287
Rabbit anti-aPKC (1:500)	Santa Cruz Biotechnology	Cat# sc-208; RRID:AB_2168668
DAPI 4′,6-diamidino-2-phenylindole (1:1000)	Thermo Fisher Scientific	Cat# D1306; RRID:AB_2629482

**Chemicals, peptides, and recombinant proteins**		

Paraformaldehyde	EMS	19340–72
Triton X-100	Euromedex	2000B
Phosphate Buffered Saline	Lonza	BE17-515F

**Experimental models: Organisms/strains**		

D.melanogaster: Aka::GFP	Byri et al., 2015^53^	N/A
D.melanogaster: hsFLP; aka^L200^, FRT40A/CyO	Esmangart de Bournonville & Le Borgne, 2020^27^	Gift from Stefan Luschnig
D.melanogaster: gli^DV3^, FRT40A/CyO	Schulte et al., 2003^86^	Gift from Vanessa Auld
D.melanogaster: *nrv2*^*K133153*^, FRT40A/CyO	Esmangart de Bournonville & Le Borgne, 2020^27^	DGRC 114351
D.melanogaster: w^∗^; UAS-Ras^V12^; M6^W186Stop^, FRT79E/TM6, Tb^1^	Dunn et al., 2018^38^	Gift from Tian Xu
D.melanogaster: hs-FLP; If/CyO; M6^W186Stop^, FRT79E/TM6, Tb^1^	Esmangart de Bournonville & Le Borgne, 2020^27^	Gift from Tian Xu
D.melanogaster: yw, eyFLP; Act > y+Gal4, UAS-GFP; tub-Gal80, FRT79E	Esmangart de Bournonville & Le Borgne, 2020^27^	Gift from Tian Xu
D.melanogaster: *hs*Flp, act-Gal4, UAS-nls:GFP/FM6; ptub-Gal80, FRT40A/CyO	Perdigoto et al., 2011^87^	
D.melanogaster: yw, eyFLP; *aka*^*L200*^, FRT40A; MKRS/SM5 – TM6, Tb, Hu	This study	N/A
D.melanogaster: yw, eyFLP; *aka*^*L200*^, FRT40A; UAS-*Ras*^*V12*^/SM5 – TM6, Tb, Hu	This study	N/A
D.melanogaster: yw, eyFLP*; gli*^*DV3*^, FRT40A; MKRS/SM5 – TM6, Tb, Hu	This study	N/A
D.melanogaster: yw, eyFLP*; gli*^*DV3*^, FRT40A; UAS-*Ras*^*V12*^/SM5 – TM6, Tb, Hu	This study	N/A
D.melanogaster: yw, eyFLP*; nrv2*^*K13315*^, FRT40A; MKRS/SM5 – TM6, Tb, Hu	This study	N/A
D.melanogaster: yw, eyFLP*; nrv2*^*K13315*^, FRT40A; UAS-*Ras*^*V12*^/SM5 – TM6, Tb, Hu	This study	N/A

**Software and algorithms**		

Fiji (version 2.14.0)	Schindelin et al., 2012^88^	https://imagej.net/Fiji
LEICA LAS AF software		RRID:SCR_013673
Imaris	Bitplane	https://imaris.oxinst.com/packages; RRID:SCR_007370
Inkscape		https://inkscape.org/
Graph Pad Prism 6		https://www.graphpad.com/
Computational model (source code)	(Plunder et al., 2024)^46^	https://github.com/SteffenPL/sEMTor.jl

**Other**		

Confocal Microscope	Leica	LSM TCS SPE, TCS SP5 and TCS SP8

### Experimental model and study participant details

#### Drosophila melanogaster

All *Drosophila* stocks were maintained on media consisting of corn meal, sugar, yeast, and agar on incubators at a constant temperature of 25°C.

### Method details

#### Larvae staging

Flies were crossed and flipped ∼48h after initial crossing to stage larvae using as reference the number of hours after egg laying (h AEL). A 2h interval was left after the flip to allow for egg deposition, taking this time point as the “0h AEL” reference.

#### Immunostaining, image acquisition and image analysis

Eye-antenna imaginal discs from staged larvae were dissected and fixed for 15min in 4% formaldehyde & permeabilized/washed in PBT 0.1%, stained with primary antibody solution for 3h at RT, washed and then stained overnight with secondary antibody solution & DAPI (0.5 mg/mL, 1:1000 dilution) at 4°C. The antibodies used are detailed in the key resources table. Dissected imaginal discs were mounted in 0.5% N-propyl-gallate dissolved in 90% glycerol/PBS 1× final & imaged on a Leica inverted confocal SP8 or a Leica upright confocal SPE using dry 20× (N.A. 0.7) and oil immersion 63× (N.A. 1.4) objectives and controlled by LAS AF software. All image processing and analysis was performed using Fiji.

#### Computational modeling

The model has been modified from a previously published model.^46^ Briefly, cells are abstracted to nuclei attached to a set of adjustable springs connecting them to an apical point and a basal point. Apical points of adjacent cells are also attached by adjustable springs. Basal points are attached to a simple non-deformable basal line representing the basement membrane. Nuclei repel each other via a repulsion force acting on their soft outer cores. The harder, inner cores of nuclei cannot overlap. Loss of apical or basal adhesion (events *A* and *B*) remove the adjustable apical-nuclei or basal-nuclei springs, respectively. Apical constriction (***ApC***) increases the strength of the strings between adjacent apical points for clone cells. Parameters were adapted to allow squamous and short pseudostratified configurations to emerge. The transition between pseudostratified and squamous epithelial epithelium is achieved by changing a limited set of parameters (see Table S2). For instance, the allowed maximal distance between basal points. When set to 10 μm this distance allows cells to arrange themselves as a squamous monolayer. When set to 3 μm it forces cells to redistribute their nuclei along the apicobasal axis to minimize tension/compression and this generates pseudo-stratification. To prevent the pseudostratified tissue from growing over 3–4 pseudo-layers a maximum height is set and acts as a hard constraint. Since by default the basal compartment located below the basal line is permissive and does not act as a physical obstacle, the squamous tissue tends to flip and rotate around the basal line. To prevent this behavior, we added a very weak repulsive force within the basal compartment which pushes nuclei weakly upwards as soon as they cross the basal line. Forces in the model have no unit and are expressed proportionally to the nuclei-nuclei repulsion force. The relative strength of the basal repulsion force is 2.5% of the nuclei-nuclei repulsion force strength and does not prevent basal delamination. For simulations with matrix resistance, we tuned this force to 250% of the nuclei-nuclei repulsion force. Further modification of the model includes a new event called ***Ai***. During this event, the apical-apical springs at the interface between control cells and clone cells are removed and a recovery phase is added where the two disconnected apical layers of control cells directly connect again with a long apical-apical spring crossing the gap caused by the intermediate EMT cells. The rest length of this apical-apical spring goes to zero with a constant speed of 5 μm/h. Montage of simulation outputs into supplementary movies was made using VSDC video editor.

### Quantification and statistical analysis

#### Clone size quantification

Clone size was quantified in eye discs stained with anti-E-cad (gray) and anti-phosphorylated histone 3 (PH3) or Cleaved-Casape-3 (C-Cas3) by manually counting GFP-positive nuclei belonging to *SJ*^−/−^*, Ras*^*V12*^ clones during delamination.

#### Quantification of apical delamination stages

The percentage of *M6*^*W186Sto*^*-Ras*^*V12*^ clones at different stages of apical delamination was performed by manually counting in individual eye discs the number of *M6*^*W186Stop*^*-Ras*^*V12*^ clones at a four specific stages: clones with no junctional remodeling, octopus stage, tissue to lumen transition and clones detached in the lumen. The number of each event (stage) was then divided by the number of clones within the eye disc. The data used for the quantification was from apical delamination events in eye discs stained with anti-E-cad and one of the following markers: Coracle, β-integrin, aPKC, cDcp1, P-Myo, F-actin. See Table S1.

#### Quantification of delamination rates

The mean and the standard error of the mean (SEM) of delamination events were calculated in each of the simulated scenario performed in single cells, in clones of various sizes, in the presence of a non-permissive basal compartment and in the absence of a non-permissive basal compartment. Each simulation scenario was performed 200 times. The results of these analyses are detailed in Figures 6E–6N and in Figures 7A–7H.
