# Supplementary material for: The directionality of collective cell delamination is governed by tissue architecture and cell adhesion in a Drosophila carcinoma model
Source: iScience. 2025 Sep 30;28(11):113663. doi: 10.1016/j.isci.2025.113663 (PMC12555851; doi:10.1016/j.isci.2025.113663)
Supplement: Document S1. Figures S1–S5, Tables S1, S2, and Data S1 [file mmc1.pdf]

iScience, Volume *28*

Supplemental information

The directionality of collective cell delamination is governed by tissue architecture and cell adhesion in a *Drosophila* carcinoma model

Marta Mira-Osuna, Steffen Plunder, Eric Theveneau, and Roland Le Borgne

Supplemental Figures

Figure S1. The loss of SJ integrity in epithelial cells triggers caspase activation and cell death. Related to Figure 1

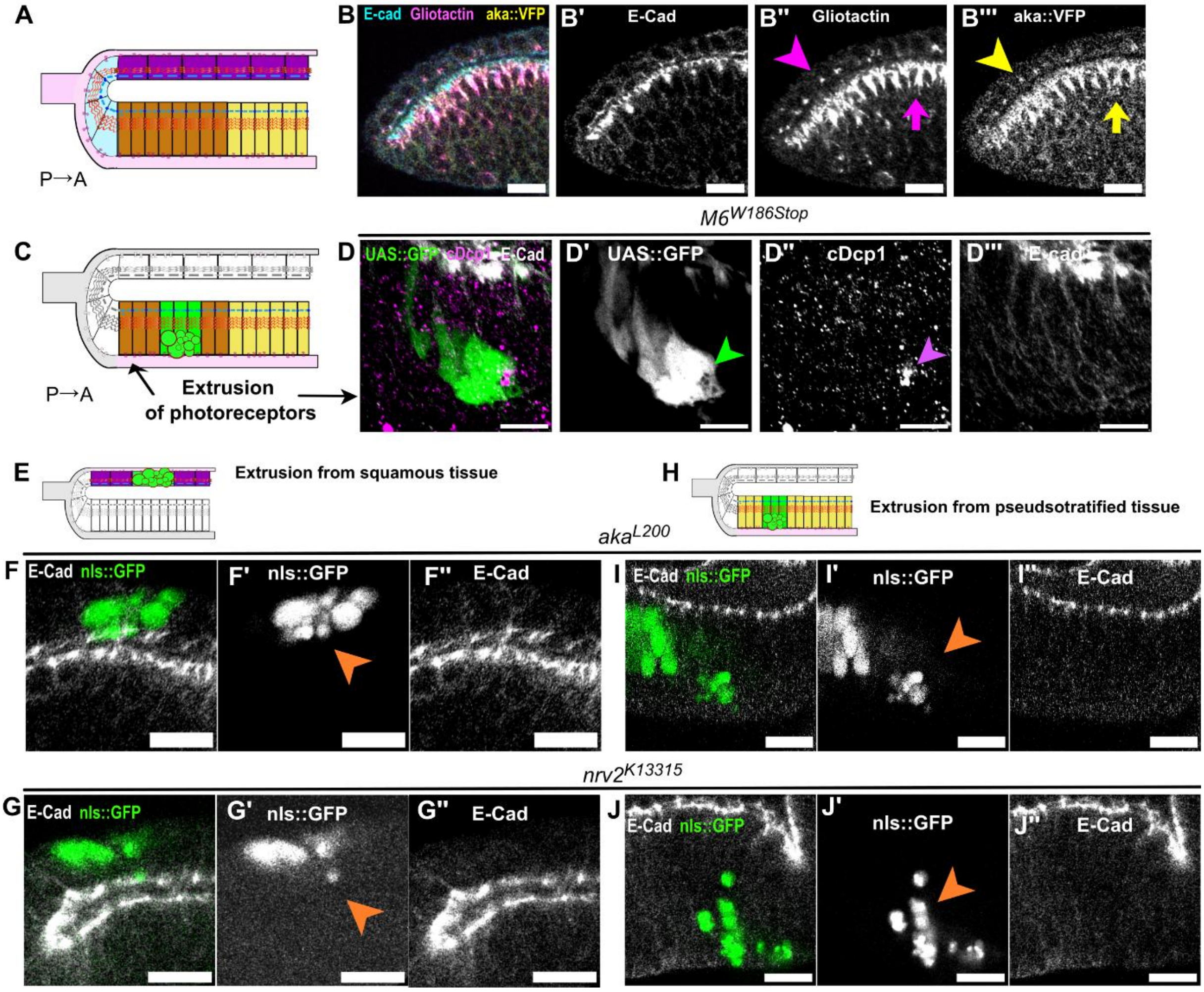

(A, C, E, H) Schemes of orthogonal sections along the dorso-ventral axis of the *Drosophila* eye disc (A) wild-type eye disc, (C) extrusion of clones posterior to the MF already differentiated in photoreceptors, (E) extrusion of clones in the squamous epithelium (H) extrusion of clones anterior to the MF in the pseudostratified epithelium. The posterior-to-anterior axis is left-to-right, the peripodial epithelium (PE) in violet, undifferentiated cells in the Disc Proper (DP) in yellow, differentiated cells in the DP in brown, adherens junctions (AJ) in blue, septate junctions (SJ) in red, focal adhesions (FA) in magenta, the extracellular matrix (ECM) in light magenta. Green cells represent clones generated using the eyMARCM system.

(B-B''') Localization of aka::VFP (yellow), E-cad (grey) and Gli (magenta) in the squamous (arrowheads) and pseudostratified (arrows) epithelia of wild type eye disc.

(D-D"''") Eye disc stained for E-cad and cDcp1. M6 clones (green arrowhead) in the differentiated part of the pseudostratified epithelium show fragmented cytoplasm cell (green arrowhead) and are caspase-activated (violet arrowhead).

(F-G'', I-J'') Eye discs stained for E-cad. Mutant cells (green) for the tricellular SJ master regulator Aka (*aka*^{L200}, F-G'') or bicellular SJ core-complex component Nrv2 (*nrv2*^{K3315}, I-J'') in the squamous (F-F'', I-I'') or pseudostratified epithelium (G-G'', J-J'') contain pyknotic bodies (orange arrowheads).

Scale bars represent 10 µm. Each image is a single confocal optical section. Posterior left, dorsal up. See Supplementary Information for exact genotypes and sample sizes.

Figure S2. The loss of SJ integrity in *Ras*^{V12}-cells leads to apical and basal collective delamination of cells alive out of the epithelium. Related to Figure 2

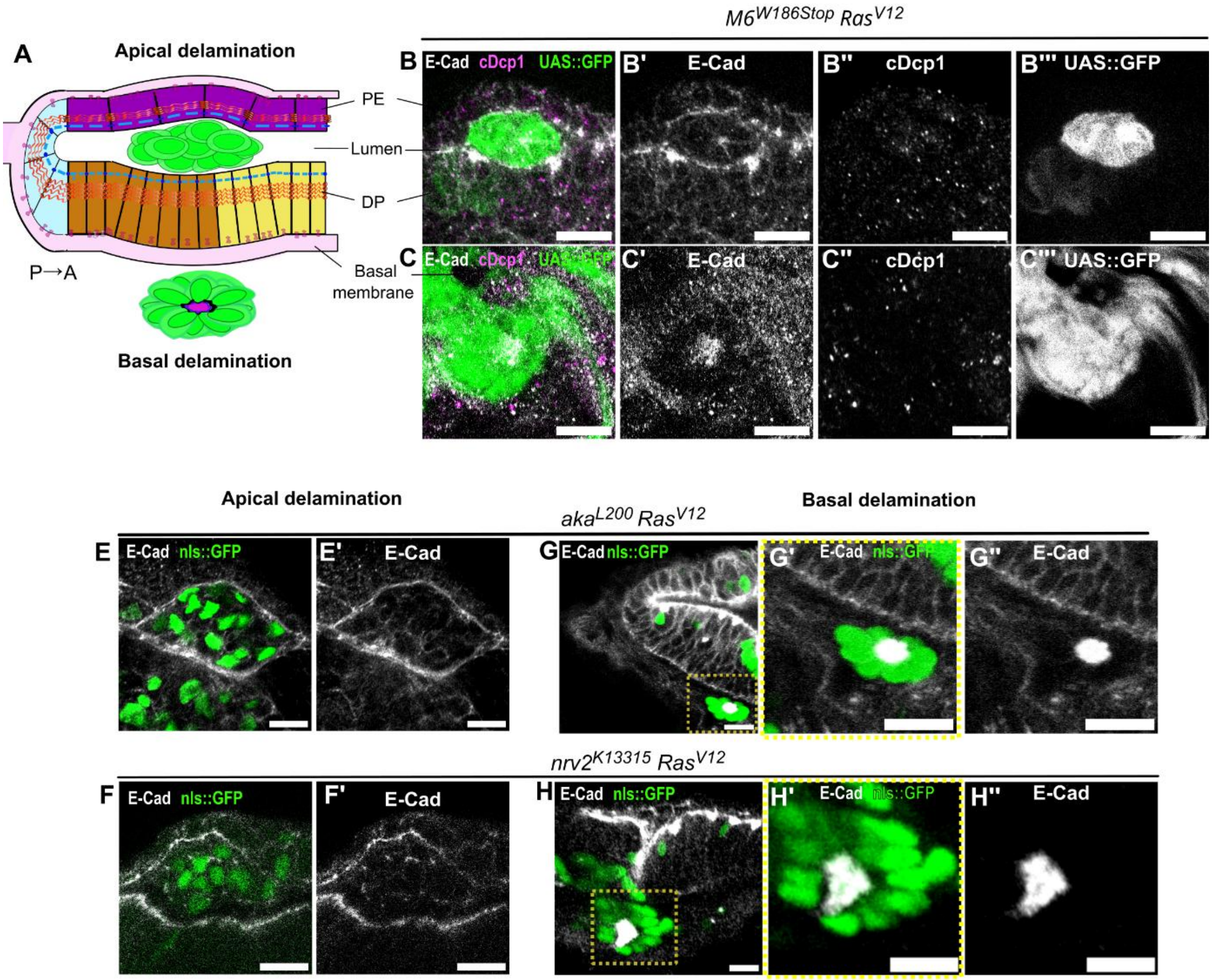

(A) Scheme of orthogonal section along the dorso-ventral axis of SJ- depleted, *Ras*^{V12} clones undergoing apical and basal collective delamination.

(B-B''', C-C''') Eye discs stained for E-cad (grey) and cDcp1 (magenta). SJ- depleted, *Ras*^{V12} clones (green) undergo collective delamination independently of caspase-activation and delaminate apically in the lumen (B) or basally forming rosettes (C).

(E-H'') Eye discs stained for E-cad (grey). *Ras*^{V12} cells depleted in a tricellular (*aka*^{L200}) or bicellular (*nrv2*^{K13315}) SJ-component (green) undergo apical (E-E', F-F4) and basal (G-G''', H-H'') collective delamination. Yellow dotted box marks inset for rosettes close up.

Scale bars represent 10 µm. Each image is a single confocal optical section. Posterior left, dorsal up. See Supplementary Information for exact genotypes and sample sizes.

Figure S3. Rosettes are composed of polarized and differentiated cells that delaminate basally from the pseudostratified epithelium. Related to Figure 4

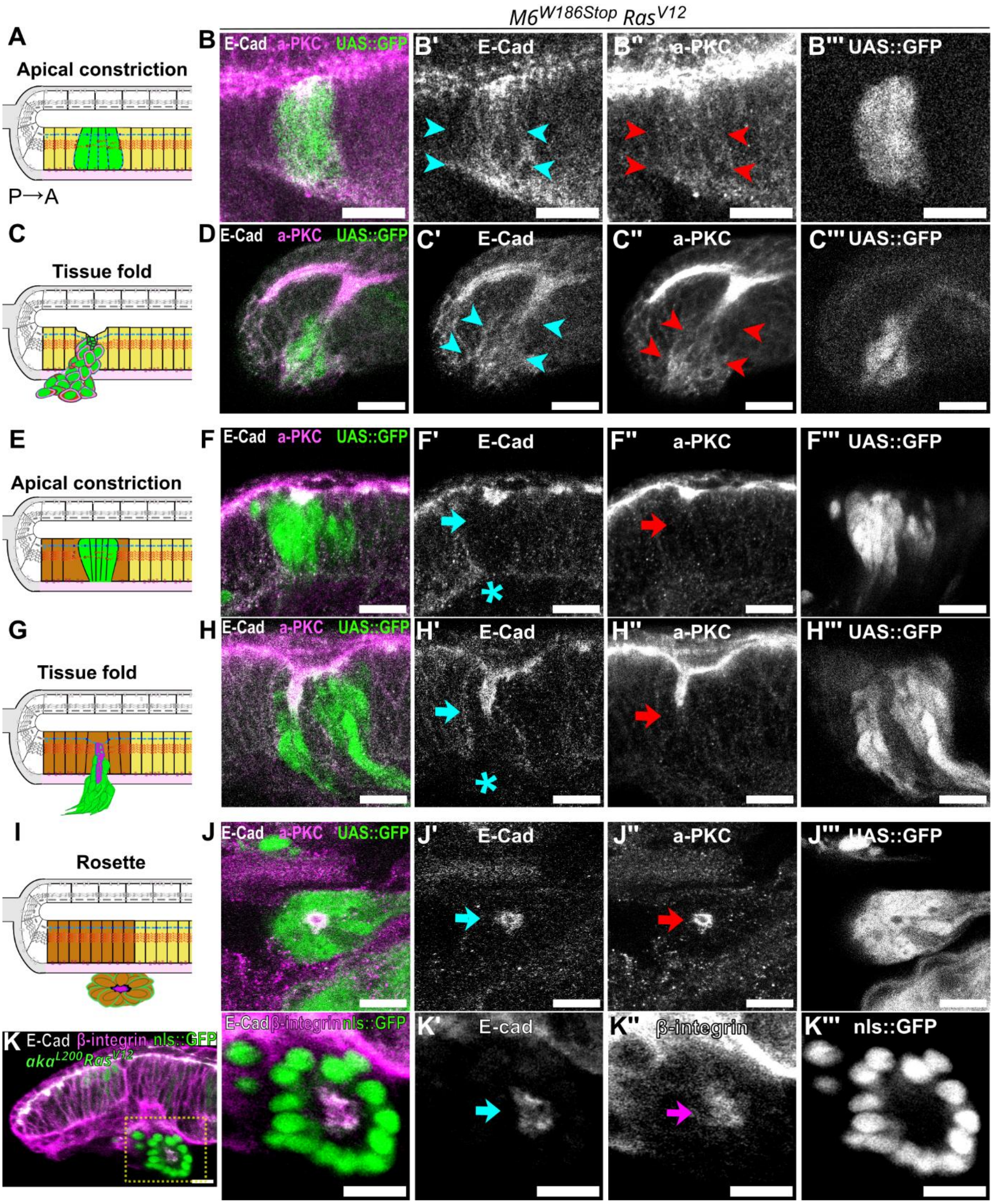

(A, C, E, G, I) Schemes of orthogonal sections along the dorso-ventral axis of the *Drosophila* eye disc with SJ-depleted, Ras^{V12} clones (green) forming tissue folds anterior (A, C, yellow) and posterior (E, G, brown) to the MF, or undergoing basal delamination to form rosettes (I, brown).

(B, D, F, H, J) Heterozygous eyes stained for E-cad (grey) and aPKC (magenta).

(B-B''', D-D''') *M6*^{W186Stop}-*Ras*^{V12} clones anterior to the MF (*M6*^{W186Stop}*Ras*^{V12}, green) constrict apically (B) and invaginate (D) concomitant to basolateral E-cad delocalization (B', C', blue arrowheads) and a partial loss of polarity, noted by the partial basal localization of aPKC (B'', C'', red arrowheads).

(F-F''', H-H''') *M6*^{W186Stop}-*Ras*^{V12} clones posterior to the MF (green) constrict apically (F) and invaginate (H) with E-cad localizing apically (F', H', blue arrow) and being lost form the basal pool (blue asterisk) concomitant to aPKC apical restriction (F'', H'', red arrows), suggesting polarity is preserved.

(J-J''') *M6*^{W186Stop}-*Ras*^{V12} clones delaminate basally and form rosettes (green) that contain polarized cells and E-cad (J', blue arrow) and aPKC (J'', red arrow) are restricted to the apical domain.

(K-K''') Eye stained for E-cad (grey) and β-integrin (magenta). *SJ*$^{-/-}$-*Ras*^{V12} clones (green) forming basally delaminated rosettes exhibit adhesion proteins at the center of the radial cluster, at the apical domain, namely E-cad (K', blue arrow) and β-integrin (K'', magenta arrow).

Scale bars represent 10 μm. Each image is a single confocal optical section. Posterior left, dorsal up. See Supplementary Information for exact genotypes and sample sizes.

Figure S4. Apical constriction does not lead to delamination but is sufficient to trigger basal displacement of cells in the pseudostratified epithelium. Related to Figure 6

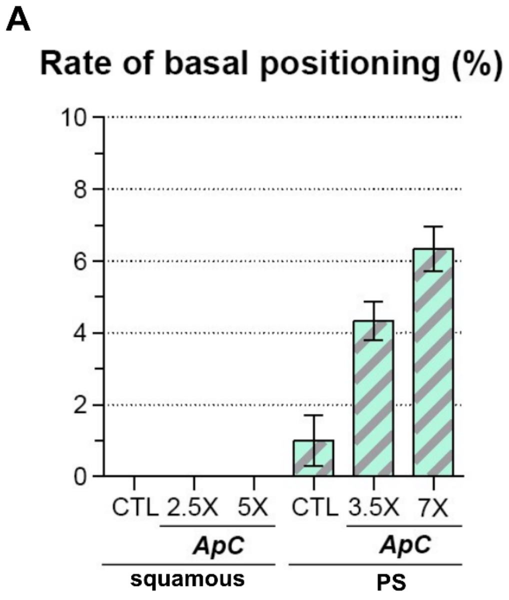

(A) Mean rate of basal positioning with standard error of the mean. To assess the effect of apical constriction, apical contractility of clone cells was increased 2.5 times (2.5X) and 5 times (5X) the control level in the squamous epithelium and 3.5 times (3.5X) and 7 times (7X) the control level in the PS epithelium. ApC, apical constriction; CTL, control levels of apical contractility; PS, pseudostratified tissue.

Figure S5. Preferential adhesion between wild-type and mutant cells at the apical neck is present during apical delamination. Related to Figure 7

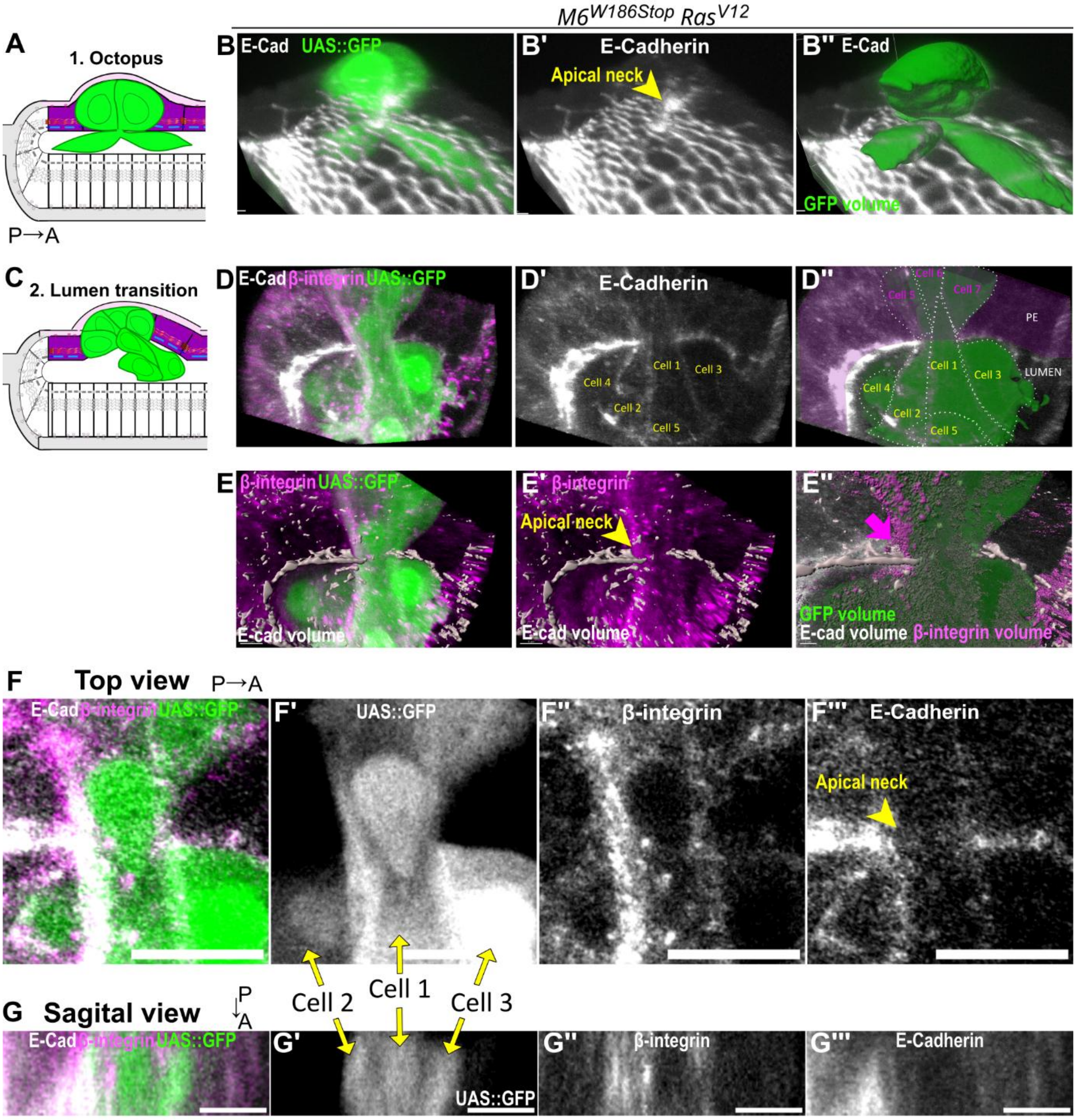

(A, C) Schematics of an orthogonal section along the dorso-ventral axis of the *Drosophila* eye disc of the squamous epithelium (purple). Mutant cells (green) apically constrict and emit lumen protrusions during the octopus stage (A) and progressively translocate into the lumen (C).
(B-B''') During apical delamination, the apical neck (yellow arrowhead) is the site where *Ras*^{V12}-cells with defective SJ integrity (*M6*^{W186Stop}-*Ras*^{V12} clone, green) emit protrusions into the lumen. Heterozygous eye disc stained with anti-E-cad (grey) was imaged and then processed with Imaris to reconstruct the volume of the GFP channel on the 3D rendering of the confocal stack. Still frames from Movie S9.

(D-G''') During the translocation of SJ-depleted *Ras*^{V12}-cells (*M6*^{W186Stop}-*Ras*^{V12} clone, green), some cells are located in the lumen (D', Cell 1-4 yellow), while other cells are still embedded in the squamous epithelium (PE) (D'', Cell 1-3 magenta). Both pools of cells belong to the same clone and can be seen connected at the apical neck (E', yellow arrowhead), which is also labeled with β-integrin (E'', pink arrow). The apical neck view with a top (F) and a sagittal (G) cross-section exhibits the constricted

nucleus of one cell that is translocating into the lumen (Cell 1) and the cytoplasm of two cells that have already translocated in the lumen (Cell 2 and Cell 3). Heterozygous eye disc stained with anti-E-cad (grey) and β-integrin (magenta) was imaged and then processed with Imaris to reconstruct the volume of the GFP channel (E'' green), the E-cad signal (E-E'', grey) and the β-integrin signal (E'', magenta) on the 3D rendering of the confocal stack. Still frames from Movie S10.

Scale bars represent 10 µm for (B-F''') and 5µm for (G-G'''). All images are single confocal optical sections with posterior to the left, anterior to the right (B-F''') and posterior top, anterior bottom (G-G'''). See Supplementary Information for exact genotypes and sample sizes.

Data S1 List of the complete genotypes for Figures and Supplemental Figures.

Figure 1
B-D)

yw, eyFlp / +; act-Gal4, UAS-nls::GFP / ; + / tub-Gal80, FRT, 79E

F-H''')

yw, eyFlp / +; act-Gal4, UAS-nls::GFP / + ; *M6*^{W186Stop}, FRT79E / tub-Gal80, FRT, 79E

J-K''')

yw, eyFlp / +; UAS-*Ras*^{V12} / act-Gal4, UAS-nls::GFP ; *M6*^{W186Stop}, FRT79E / tub-Gal80, FRT, 79E

Figure 2
B-C-E-F-H-I)
yw, eyFlp / +; UAS-*Ras*^{V12} / act-Gal4, UAS-nls::GFP ; *M6*^{W186Stop}, FRT, 79E / tub-Gal80, FRT, 79E

Figure 3
B-C-E-F)
yw, eyFlp */ hsFlp,* act-Gal4, UAS-nls::GFP ; *aka*^{L200}, FRT40A / ptub-Gal80, FRT, 40A ; UAS-*Ras*^{V12} / +

Figure 4
B-D-F-H)
yw, eyFlp / +; UAS-*Ras*^{V12} / act-Gal4, UAS-nls::GFP ; *M6*^{W186Stop}, FRT, 79E / tub-Gal80, FRT, 79E
J)
yw, eyFlp */ hsFlp,* act-Gal4, UAS-nls::GFP ; *aka*^{L200}, FRT40A / ptub-Gal80, FRT, 40A ; UAS-*Ras*^{V12} / +

Figure 5
B-C-D-E-G-I-J)
yw, eyFlp / +; UAS-*Ras*^{V12} / act-Gal4, UAS-nls::GFP ; *M6*^{W186Stop}, FRT, 79E / tub-Gal80, FRT, 79E

Figure 7
J-L-N)
yw, eyFlp / +; UAS-*Ras*^{V12} / act-Gal4, UAS-nls::GFP ; *M6*^{W186Stop}, FRT, 79E / tub-Gal80, FRT, 79E

Figure S1
B)

yw ; Aka-VFP/ CyO ; +/+

D)

yw, eyFlp / +; act-Gal4, UAS-nls::GFP / + ; *M6*^{W186Stop}, FRT79E / tub-Gal80, FRT, 79E

F, I)

yw, eyFlp */ hsFlp,* act-Gal4, UAS-nls::GFP ; *aka*^{L200}, FRT40A / ptub-Gal80, FRT, 40A ;
G, J)
hsFlp, act-Gal4, UAS-nls::GFP /+ ; *nrv2*^{K13315}, FRT40A / ptub-Gal80, FRT, 40A ;

Figure S2
B-C)

yw, eyFlp / +; UAS-*Ras*^{V12} / act-Gal4, UAS-nls::GFP ; *M6*^{W186Stop}, FRT, 79E / tub-Gal80, FRT, 79E

E, G)
yw, eyFlp */ hsFlp,* act-Gal4, UAS-nls::GFP ; *aka*^{L200}, FRT40A / ptub-Gal80, FRT, 40A ; UAS-*Ras*^{V12} / +

F, H)
hsFlp, act-Gal4, UAS-nls::GFP /+ ; *nrv2*^{K13315}, FRT40A / ptub-Gal80, FRT, 40A ; UAS-*Ras*^{V12} /+

Figure S3
B-D-F-H-J)

yw, eyFlp / +; UAS-*Ras*^{V12} / act-Gal4, UAS-nls::GFP ; *M6*^{W186Stop}, FRT, 79E / tub-Gal80, FRT, 79E

K)
yw, eyFlp / *hsFlp,* act-Gal4, UAS-nls::GFP ; *aka*^{L200}, FRT40A / ptub-Gal80, FRT, 40A ; UAS-*Ras*^{V12} / +

Table S1. Quantification of apical delamination stages across time. Related to Figure 5K.

**Stage of apical delamination**	**Late L2** (4 eye discs, 5 clones)	**L3** (79 eye discs, 84 clones)	**Late L3** (116 eye discs, 189 clones)
No junctional remodeling	4	28	11
Octopus	1	35	94
Tissue-lumen transition	0	21	49
Delaminated in lumen	0	0	35

Table S2. Model parameters for the flat and pseudostratified tissue. Related to Figure 6

	flat tissue	PS	
key	value	value	unit
sim.t_end	80	80	hours
sim.dt	0.05	0.05	hours
duration_G2	0.5	0.5	hours
duration_mitosis	0.5	0.5	hours
life_span.min	24	10	hours
life_span.max	28	14	hours
epi.init_basal_junction_dist	1.5	1.5	R_soft
epi.init_apical_junction_dist	1.5	1.5	R_soft
epi.max_cytoskeleton_length	na	3.2	R_soft
R_hard	0.3	0.3	R_soft
R_soft	5	5	μm
cytoskeleton_init	1.5	1.5	R_soft
apical_junction_init	0.333	0.3333	R_soft
max_basal_junction_dist	2	0.6	R_soft
epi.mu	0.1	0.5	hours/R_soft
epi.k_apical_healing	-0.2	-0.2	1/hours
apical_cytos_strain	contract	relax	n.a.
basal_cytos_strain	contract	relax	n.a.
basal_damping_ratio	0.5	1	a.u.
diffusion	0.1	0.1	a.u.
k_apical_junction	-5	-0.01	1/hours
k_cytoskeleton	5	5	1/hours
stiffness_apical_apical	5	8	a.u.
stiffness_apical_apical (for local apical constriction)	12.5	28	a.u.
stiffness_nuclei_apical	2	2	a.u.
stiffness_nuclei_basal	2	2	a.u.
stiffness_repulsion (between soft cores)	4	4	a.u.
stiffness_straightness	15	15	a.u.
basal_repulsion (default/permissive)	0.1	0.1	a.u.
basal_repulsion (non-permissive)	10	10	a.u.

The parameters that differ between the two configurations are highlighted in grey. Stiffness and repulsion factors have no units it is their relative strength that matters. Size and distances are expressed as a function of R_soft (radius of the nucleus soft core). a.u, arbitrary unit; n.a., not applicable.
